# Beyond Body Weight: A Comprehensive Review of Allometric Scaling in Drug Development for Human Dose Predictions

**DOI:** 10.3390/pharmaceutics18070824

**Published:** 2026-07-03

**Authors:** Marlon C. Mallillin, Daniela A. Silva, Neil A. Miller, Shengnan Zhao, Maryam Salami, Raimar Löbenberg, Neal M. Davies

**Affiliations:** 1 Faculty of Pharmacy and Pharmaceutical Sciences, University of Alberta, Edmonton, AB T6G 2H7, Canada; mallilli@ualberta.ca (M.C.M.III); shengna2@ualberta.ca (S.Z.); msalami2@ualberta.ca (M.S.); 2 Department of Pharmacy, Faculty of Pharmacy, University of Santo Tomas, Manila 1015, Philippines; 3 Simulations Plus, Inc., Lancaster, CA 93534, USA; daniela.silva@simulations-plus.com (D.A.S.); neil.miller@simulations-plus.com (N.A.M.); 4 China Z. Pharmaceutical Productivity Centre, Beijing 101111, China; 5 Department of Food Science, Engineering and Technology, College of Agriculture and Natural Resources, Karaj Campus, University of Tehran, Karaj 31587-77871, Iran

**Keywords:** allometric scaling, pharmacokinetics, pharmacodynamics, drug development, first-in-human (FIH), PBPK modelling, Kleiber’s law, maximum recommended starting dose (MRSD), minimum anticipated biological effect level (MABEL), intestinal lymphatic transport, lysosomal sequestration, in vitro–in vivo extrapolation (IVIVE)

## Abstract

Allometric scaling provides a practical framework for predicting human pharmacokinetic (PK) parameters from animal data by relating physiological processes to body size through power-law equations. Despite its simplicity and widespread use in first-in-human (FIH) dose selection, its predictive performance is limited by species-specific differences in absorption, distribution, metabolism, and excretion (ADME). This review summarizes the mathematical foundations, workflows, and diagnostics of allometric scaling, while critically examining where the approach succeeds and where it fails. Core concepts, including clearance, volume of distribution, correction factors, and the rule of exponents, are discussed alongside complementary methods: in vitro–in vivo extrapolation (IVIVE), physiologically based pharmacokinetic (PBPK) modelling, and the Wajima normalized time-course method. Historical clinical failures, including fialuridine, TGN1412, BIA 10-2474, and rofecoxib, illustrate the limits of relying solely on allometry, while thalidomide and the fenfluramine combination exemplify toxicodynamic species-selection failures. Modern advances, including the Extended Clearance Classification System (ECCS), target-mediated drug disposition, FcRn recycling, and emerging artificial intelligence and machine-learning methods, are integrated within a framework. Overall, the review treats allometric scaling as a disciplined starting hypothesis that must be triangulated with mechanistic, experimental, and regulatory evidence to support safer and more reliable human translation.

## 1. Introduction: Rationale for Allometric Scaling

The foundation of allometric scaling in drug development rests on a century of observations that many physiological processes scale sublinearly with organismal mass. The concept traces to Rubner’s surface law of the late nineteenth century. Still, it was Kleiber who provided the seminal quantitative synthesis, demonstrating that basal metabolic rate (BMR) scales with body mass to approximately three-quarters of a power law [1,2]. This relationship is expressed as:

*B* = *aM*^0.75^ (1)

where *B* is the basal metabolic rate, *M* is body mass, and *a* is a proportionality constant that varies among taxa. The apparent universality of this relationship across organisms spanning many orders of magnitude in body mass, from unicellular organisms to large mammals, has fascinated biologists and pharmacologists alike.

West, Brown, and Enquist [3,4] developed a network-based model to explain the mechanistic origin of Kleiber’s three-quarter power law. Their framework demonstrated that the geometry and efficiency of space-filling vascular networks, combined with size-invariant terminal units (e.g., capillaries) and energy-minimizing transport systems, naturally yield quarter-power exponents across biological scaling relationships. This theoretical grounding elevated allometric scaling from an empirical curiosity to a principle with biophysical underpinning, though debate about the precise exponent value continues [5,6]. The exponent itself remains contested: dimensional and surface-area arguments predict a value closer to 0.67, and a reanalysis of large mammalian datasets found no support for a universal three-quarter exponent, with empirical estimates for individual physiological and pharmacokinetic parameters typically falling between roughly 0.67 and 0.80 [7,8]. For interspecies pharmacokinetic scaling, the practical implication is that 0.75 should be treated as a convenient default rather than a biophysical constant, since clearance projections are sensitive to the exponent assumed.

In drug development, pharmacokineticists adopted this logic to extrapolate pharmacokinetic (PK) parameters, including clearance (CL), volume of distribution (V_D_), and half-life (t½), across species [5,6,9,10,11]. The mathematical and physiological foundations of these parameters, articulated in the canonical textbook treatment of Rowland and Tozer [12], underpin all of the operational moves that follow. Dedrick’s pioneering analyses of interspecies time-scaling and methotrexate disposition in the early 1970s [13] supplied the conceptual bridge between Kleiber-style biological allometry and quantitative pharmacokinetic extrapolation, anticipating later profile-superposition methods that are now formalized in the Wajima approach (Section 6). Operationally, the approach involves fitting the allometric equation, Y = a(BW)^b^, where Y is the pharmacokinetic parameter of interest, BW is the body weight, a is the allometric coefficient, and b is the allometric exponent. Logarithmic transformation yields ln(Y) = ln(a) + b·ln(BW), allowing linear regression across multiple species and projection to a 70 kg human reference weight. As a rapid, auditable method, allometry remains attractive in early development; however, the apparent universality of the allometric exponent can obscure compound-specific ADME determinants that differ markedly across species [6,14]. For practical orientation to species and starting exponents used in development programs, Table 1 summarizes typical applications and cautions by species.

### Scope, Novelty, and Approach

Several authoritative reviews have addressed individual elements of interspecies translation. Still, they have tended to treat empirical allometry, physiologically based pharmacokinetic (PBPK) modelling, clearance classification, biologic disposition, and emerging computational methods as largely separate studies. The contribution of the present review is integrative rather than encyclopedic: it draws classical body-weight allometry, the rule of exponents, in vitro–in vivo extrapolation (IVIVE), PBPK modelling, the Extended Clearance Classification System (ECCS), target-mediated drug disposition, intestinal lymphatic transport, lysosomal sequestration, and machine-learning approaches into a single, mechanism-anchored framework for first-in-human pharmacokinetic and dose prediction. By positioning allometry as a disciplined starting hypothesis to be triangulated against these complementary methods, and by pairing the methodological account with worked examples and a structured decision path, the review is intended to function as a practical planning instrument rather than a survey of any one technique.

A unifying way to read the methods that follow is as a hierarchy of prediction reliability, in which mechanistic resolution and data requirements both increase at each step. Level 1 is body-weight allometry: fast, transparent, and best used as a hypothesis generator. Level 2 is corrected allometry, in which the rule of exponents and maximum-life-span or brain-weight terms repair systematic bias in clearance projection. Level 3 is mechanistic in vitro–in vivo extrapolation, which resolves species differences in enzyme and transporter activity. Level 4 is physiologically based and target-mediated modelling, namely PBPK, target-mediated drug disposition, and quantitative systems pharmacology, which integrates system physiology and supports special populations, drug interactions, and regulatory submissions. The central thesis of this review is that allometry should anchor the top of this ladder as a disciplined starting hypothesis, and that a programme should climb only to the level justified by the compound’s mechanism and the stakes of the decision, rather than defaulting to either the simplest or the most elaborate method.

This is a narrative, non-systematic review. Sources were selected for their conceptual and methodological relevance to interspecies pharmacokinetic scaling and first-in-human dose prediction, with emphasis on primary methodological papers, illustrative clinical case histories, and current regulatory guidance, rather than through a registered systematic review protocol. The scope is deliberately mechanistic and translational; it does not attempt an exhaustive enumeration of every published allometric analysis, and the worked examples are hypothetical constructs chosen for didactic clarity.

## 2. Building the First Empirical Bridge

The typical allometric workflow begins with PK data generated experimentally in preclinical species (commonly mouse, rat, dog, and non-human primate) [5,6,16]. For each parameter *Y* of interest, an ordinary least squares (OLS) regression of ln(Y) on ln(BW) yields a slope *b* and a scale factor *a*, as detailed below. Extrapolation to a 70 kg reference human with appropriate prediction intervals provides initial estimates of CL, V_D_, and t½ that bound candidate doses [10,11]. Because three to four species are typical and uncertainty is substantial, empirical fits should be triangulated against mechanism-aware IVIVE and PBPK model estimates to ensure robustness [14,22,23].

### 2.1. Mathematical Framework

Allometric scaling is predicated on the power-law relationship CL = *a*(BW)*^b^*, where BW is body weight and *a* and *b* are the coefficients and exponent of the allometric equation, respectively. Log-linearization transforms this to log_10_(CL) = log_10_(*a*) + *b*·log_10_(BW), enabling OLS regression in log space, where log_10_(*a*) is the y-intercept and *b* is the slope [5,10]. This linearization, while convenient, introduces weighting bias because body weights span orders of magnitude, and single-species idiosyncrasies can rotate the regression slope [24].

### 2.2. Worked Example: Allometric Prediction of Clearance

To illustrate the operational workflow, consider a hypothetical drug with clearance data from four preclinical species (Table 2). The objective is to predict human clearance using simple allometric scaling.

**Figure 1:**
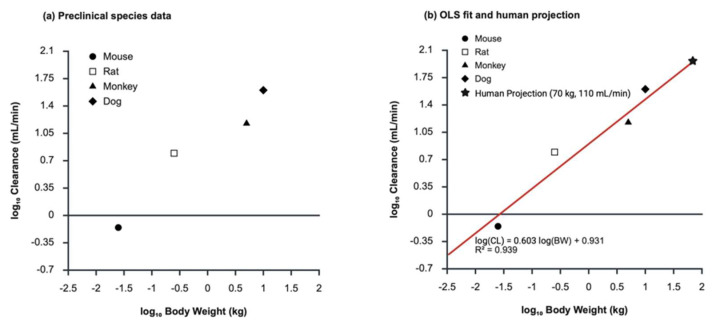
Allometric scaling of clearance for the hypothetical drug. (**a**) Raw log–log scatter of the four preclinical species (mouse, rat, monkey, dog), shown without overlay so the empirical dispersion can be judged independently of any model. (**b**) The same data with the ordinary least squares (OLS) regression line log_10_(CL) = 0.603 × log_10_(BW) + 0.931, R^2^ = 0.939 and the projected human clearance at 70 kg (110 mL/min, star) overlaid.

Converting body weight and clearance to log_10_ scale and performing OLS regression yields the equation log_10_(CL) = 0.603 × log_10_(BW) + 0.931. Substituting the human body weight of 70 kg (log_10_ [25] = 1.845) gives log_10_(CL) = 0.603 × 1.845 + 0.931 = 2.043. Converting back: CL = 10^2.043^ ≈ 110 mL/min. This predicted human clearance of approximately 110 mL/min serves as an initial estimate that must then be contextualized against mechanistic data [6,14]. The clearance values used here are illustrative inputs for the OLS regression worked example; the Wajima worked example in Section 6.2, and Appendix A.2 employs an independent hypothetical parameter set selected to illustrate the superposition principle, and the two examples are not numerically linked. Although the fit looks convincing (R^2^ = 0.939), the human projection lies far outside the fitted body-weight range. Hence, its statistical uncertainty is large: the 95% confidence interval for the mean prediction spans roughly 13 to 905 mL/min, and the 95% prediction interval spans roughly 6 to 1970 mL/min (Appendix A.1.5), a concrete illustration of the point made in Section 2.3 that a high R^2^ does not constrain extrapolative reliability. Figure 1 presents this worked example graphically, contrasting the raw interspecies scatter (Figure 1a) with the fitted OLS line and the projected human clearance at 70 kg (Figure 1b).

### 2.3. Limitations of R^2^ in Allometric Extrapolation

A common but misleading practice is to rely on the coefficient of determination (R^2^) as a measure of confidence in allometric human predictions. R^2^ reflects goodness of fit within the observed preclinical data range but does not quantify predictive reliability when extrapolating to human body weight, which lies outside the fitted range [6,24]. A high R^2^ can coexist with poor human predictive performance if species-specific metabolic pathways diverge at larger body weights. In contrast, a low R^2^ does not necessarily preclude acceptable extrapolative accuracy if the overall model structure is mechanistically sound.

Superior alternatives for assessing confidence in allometric predictions include: (i) prediction intervals and credible intervals for the allometric exponent and projected human parameter [6]; (ii) leave-one-species-out cross-validation, which tests model robustness by systematically excluding each species and comparing predictions [5]; (iii) biological plausibility constraints informed by known physiological scaling laws and mechanistic models [3,21]; and (iv) model-selection criteria such as the Akaike Information Criterion (AIC) or Bayesian Information Criterion (BIC) to balance fit quality with model complexity [26].

## 3. Mathematics and Diagnostics of the Log–Log Line

Log-linearization makes power-law relationships tractable for least-squares regression, but several data features require discipline and careful interpretation [6,24]. First, the weighting bias inherent in log transformation means that body weights spanning orders of magnitude can disproportionately rotate the regression slope due to single-species idiosyncrasies. For example, a rat exhibiting unusually high clearance driven by rat-specific CYP isoforms (e.g., CYP2C11) or by greater hepatic blood flow per kg can pull the regression slope upward, generating an apparent allometric exponent of approximately 0.85 or greater and overestimating the human clearance projection. Conversely, an outlier dog with restricted biliary excretion can flatten the slope toward 0.55, leading to a downward bias in the predicted human exposure. In both cases, removing the outlier in a leave-one-species-out analysis reveals that the projection depends on a single mechanistically distinct species [6,24]. Second, curvature and heteroscedasticity in residuals are common, reflecting transitions between flow-limited and capacity-limited clearance states or the onset of metabolic saturation [14]. Third, with only three to four species typically available, the 70 kg prediction intervals are necessarily wider than the point estimates alone would suggest [6,24].

Accordingly, robust diagnostic practices should accompany every allometric fit: leave-one-species-out slopes, visual predictive checks with 95% confidence and prediction bands, prospective error quantification, and parallel consultation of IVIVE and PBPK results to determine whether measured binding, intrinsic clearance (which dominates the allometry of clearance), or uptake and efflux transporters (which can influence the allometry of both clearance and volume of distribution) explain outlier species. Recognizing that intrinsic clearance and transporters scale differently with body size and that they affect distinct PK parameters is essential when interpreting interspecies extrapolations [14,26].

**Figure 2:**
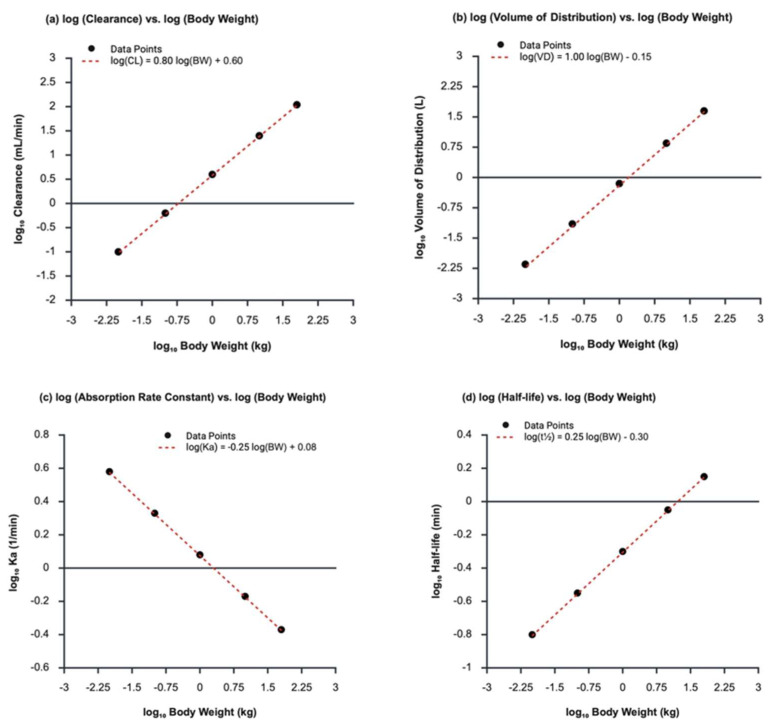
Representative log–log scaling relationships for key PK parameters. (**a**) Clearance versus body weight on a log–log scale. The slope approximates 0.75, consistent with Kleiber’s law for metabolic rate, but species expressing unique CYP isoforms may deviate. (**b**) Volume of distribution versus body weight (log–log). The exponent typically approaches 1.0, reflecting that body size is linearly related to drug distribution space. Highly lipophilic or tissue-binding compounds may show marked deviations. (**c**) Absorption rate constant (k_a_) versus body weight (log–log). The negative slope reflects longer gastrointestinal transit times in larger species. Interspecies variability in gastric pH, transporter expression, and first-pass metabolism limits the predictive power of this relationship. (**d**) Half-life versus body weight (log–log). Since t½ = 0.693 × V_D_/CL, prediction accuracy depends on the fidelity of both clearance and volume estimates. Crucially, these diagnostics certify only the quality of a fit within the observed range; they cannot reveal whether the underlying disposition biology is conserved across species, which is exactly where body-weight scaling most often breaks down.

These interspecies relationships are summarized in Figure 2, which plots clearance, volume of distribution, the absorption rate constant, and half-life against body weight on log–log axes. The two parameters most often used to anchor an allometric projection have characteristic slopes with a clear physiological origin. Clearance scales with body weight raised to an exponent close to 0.75, mirroring the three-quarter-power scaling of basal metabolic rate described by Kleiber and rationalized by the fractal, space-filling geometry of vascular transport networks (Section 1), because clearance is constrained by organ blood flows and metabolic capacity that increase sublinearly with body mass [1,2,3,4]. Volume of distribution, in contrast, scales with an exponent near 1.0, reflecting that the anatomical spaces into which a drug distributes, principally body water and tissue mass, increase in approximately direct proportion to body size; pronounced departures from unity therefore flag compounds whose distribution is governed by lipophilic partitioning or tissue binding rather than by bulk physiological volume [6,26].

## 4. Where Size-Based Scaling Breaks

Allometry provides a first approximation, but ADME processes are fundamentally pathway- and species-dependent, and a visually compelling log–log line across a narrow species panel may still catastrophically mispredict human pharmacokinetics [6,14,21]. The following subsections detail the mechanistic sources of failure.

### 4.1. Absorption and Bioavailability

Oral bioavailability varies with gastric pH, gastric emptying rate, intestinal transit time, bile flow composition, transporter expression (e.g., P-glycoprotein, organic anion-transporting polypeptides), and first-pass metabolism. These factors differ substantially across preclinical species, and no single allometric exponent can capture their combined effects on oral absorption [15,27]. Dogs, for example, lack N-acetyltransferase 2 (NAT2) activity, and rabbit cecotrophy alters enteric absorption (see Table 1 note); both confound extrapolation of oral PK [6,18,26].

A fundamental distinction in absorption, yet frequently overlooked in interspecies scaling, is that an orally absorbed drug reaches the systemic circulation via two parallel routes. Most compounds are absorbed across the enterocyte into the portal vein and traverse the liver before reaching the systemic circulation and are therefore subject to hepatic first-pass metabolism. A subset, however, associates with the triglyceride-rich lipoproteins assembled during lipid absorption and is carried instead into the intestinal lymphatics, entering the systemic circulation at the thoracic duct, and so bypassing the liver on first pass [28,29]. Conventional absorption and bioavailability assessments, and the body-weight regressions built upon them, implicitly assume the portal route and seldom quantify the lymphatic contribution, even though, for suitable compounds, it can represent a substantial fraction of systemic exposure [29,30]. This omission is becoming less defensible. Lipid-based delivery systems, including self-emulsifying and self-microemulsifying formulations, are increasingly deployed to improve the absorption of poorly water-soluble candidates and can deliberately recruit the lymphatic route, with attendant reductions in first-pass metabolism. At the same time, medicinal chemistry continues to generate lymphotropic drugs and prodrugs designed to exploit this pathway, whether for first-pass avoidance or for targeting of lymph-resident disease [29,31,32]. The interspecies consequences of the lymphatic route, which scales quite differently from portal absorption, are considered in Section 4.6.

### 4.2. Distribution

Volume of distribution depends on lipophilicity, plasma protein binding, tissue partitioning, and active uptake/efflux mechanisms. While the allometric exponent for V_D_ commonly approximates 1.0, certain highly lipophilic or tissue-binding drugs, such as amiodarone [33] and digoxin [34], exhibit volumes that are substantially higher than predicted. Species-specific differences in plasma protein binding (e.g., α_1_-acid glycoprotein concentrations) or tissue affinity can shift the human estimate far from the allometric prediction [35].

A specific, frequently underappreciated mechanism underlying several of these distribution outliers is lysosomal trapping. Lipophilic weak bases of suitable pKa cross membranes in their neutral form and become protonated within the acidic lumen of the lysosome, at a pH of approximately 4.5 to 5.0, where the charged species cannot readily diffuse back out and is sequestered by pH partitioning [36]. Because lysosomes are present in most cell types, this ion-trapping mechanism, together with binding to acidic phospholipids, can produce very large tissue-to-plasma partitioning and hence the high volumes of distribution and often prolonged terminal half-lives characteristic of cationic amphiphilic drugs; amiodarone, noted above as a vertical allometry outlier, is a prototypical example [36,37]. Simple body-weight scaling of volume cannot represent this behavior because its determinants, namely lysosomal abundance, luminal pH, and the lysosomal volume fraction of each tissue, do not scale cleanly with body mass and differ across tissues and species.

As with hepatic uptake and lymphatic transport, the appropriate response is mechanistic rather than allometric. The tissue composition models that underpin volume prediction in PBPK, developed by Rodgers and colleagues, capture ionization and acidic phospholipid binding but tend to underpredict the distribution of basic drugs into lysosome-rich tissues such as the lung [38,39]. Extensions that incorporate lysosomal sequestration explicitly improve these predictions yet remain incompletely validated: accounting for lysosomal pH and lysosomal volume fraction only partially resolved the lung discrepancy, and the systematically estimated lysosomal volume fractions, ranging from about 0.03% of cell volume in adipose tissue to several percent in spleen, are currently rat values, leaving the corresponding human and large-animal parameters poorly defined [39,40]. The consequences extend beyond volume of distribution: by raising the sequestered intracellular fraction at the expense of cytosolic unbound drug, lysosomal trapping perturbs the unbound intracellular concentration that drives metabolism and transport, and can therefore bias IVIVE of clearance as well, linking this mechanism to the arguments of Section 4.3 and Section 4.4 [37,40]. The issue is of growing practical importance, because a large share of basic lipophilic candidates is affected and lysosomotropism is now actively engaged with in development, whether to be avoided in compounds prone to phospholipidosis or deliberately exploited in lysosomotropic antimalarials and in oncology agents that target the altered lysosomal pH of tumor cells [36,37]. Lysosomal trapping is thus a further area in which credible interspecies translation requires explicit modelling, better human system parameters, and dedicated awareness, rather than reliance on a body-weight exponent.

### 4.3. Metabolism and Elimination

Clearance reflects enzyme kinetics (CYP, UGT, and other metabolizing enzymes), transporter expression, and organ blood flows. Human-specific cytochrome P450 polymorphisms, such as CYP2C19-mediated omeprazole metabolism [19] and CYP2C9-dependent warfarin elimination [20], can cause human clearance to deviate markedly from allometric predictions. Similarly, tacrolimus illustrates how complex binding and metabolism jointly shape both CL and V, creating compound-specific challenges for scaling [17]. The Extended Clearance Classification System (ECCS) provides a useful framework for predicting dominant clearance mechanisms and thereby assessing the likely reliability of allometric scaling for a given compound [41]. Because the ECCS framework also applies to renal filtration and transporter-mediated hepatic uptake and biliary excretion, in addition to CYP-mediated metabolism, it is treated as a standalone section (see Section 11) rather than as a subsection of metabolism; the cross-reference is provided here for readers focused on metabolic clearance.

### 4.4. Scaling of Transporter-Mediated Disposition

For drugs where hepatic uptake via organic anion-transporting polypeptides (OATPs) is rate-limiting, simple allometric scaling of total clearance may fail because transporter expression levels and substrate specificities differ between preclinical species and humans. In such cases, IVIVE incorporating transporter kinetics, or mechanistic PBPK models with explicit sinusoidal uptake modules, provides superior predictions [14,26,42]. Bosentan illustrates this failure mode in concrete terms: hepatic uptake mediated by OATP1B1 and OATP1B3 is the rate-determining step for its disposition, and the same transporter-level mechanism underlies its clinically significant interactions with cyclosporin A, rifampicin, and sildenafil [43]. Rosuvastatin provides a complementary example, with OATP1B1-mediated hepatic uptake governing systemic exposure and accounting for the marked increases in exposure observed in carriers of reduced-function SLCO1B1 polymorphisms [44]. In both cases, the species-to-human disconnect at the transporter level is invisible to a body-weight regression, and IVIVE or PBPK approaches that explicitly parameterize hepatic uptake are essential for credible human projections.

Efflux and renal transporters extend the same caution. P-glycoprotein (P-gp/ABCB1) and breast cancer resistance protein (BCRP/ABCG2) govern intestinal absorption, biliary excretion, and central nervous system penetration, and their expression, tissue localization, and substrate affinity differ appreciably between rodents, dogs, non-human primates, and humans; body-weight scaling captures none of this, whereas the Extended Clearance Classification System and related transporter-aware frameworks make the dominant process explicit [41,45]. On the renal side, the organic cation transporter OCT2 (SLC22A2) together with the multidrug and toxin extrusion proteins MATE1 and MATE2-K mediate active secretion of hydrophilic cations; metformin is the archetype, its clearance being governed by OCT2-mediated uptake and MATE-mediated efflux rather than by glomerular filtration alone, so its disposition tracks transporter activity rather than body weight [41,46]. The practical rule across uptake, efflux, and secretory transporters is identical. When a transporter is rate-determining, interspecies prediction should proceed via transporter-informed IVIVE or PBPK rather than a body-weight regression.

### 4.5. Biologics and Target-Mediated Drug Disposition

For monoclonal antibodies (mAbs) and other large biologic therapeutics, simple allometric scaling requires substantial adaptation [26,47]. The conventional exponents differ from those for small molecules: clearance typically scales with body weight to the power of approximately 0.8 to 0.9, while the central-compartment volume of distribution scales close to 1.0, reflecting the confinement of antibodies to plasma and interstitial fluid rather than their partitioning into tissues [26]. When only one or two species are available, as is common for biologics, these exponents are typically fixed to consensus values rather than estimated, mirroring the fixed-exponent single-species approach for small molecules described in Section 5.3 [48,49]. More importantly, target-mediated drug disposition (TMDD) introduces a saturable, dose- and concentration-dependent component of clearance that no power-law regression can capture. In TMDD, binding to the pharmacological target itself contributes meaningfully to elimination [50]. At low concentrations, target-mediated clearance can dominate; at high concentrations, the target is saturated and linear, FcRn-mediated elimination dominates [47,50]. Species differences in target expression, target turnover, and FcRn affinity for antibody Fc regions further complicate interspecies translation. Immunogenicity adds a third source of variability through anti-drug antibody formation, which can unpredictably accelerate clearance in human studies even when preclinical species tolerate the molecule well [47]. A practical constraint in biologics first-in-human modelling is that not all of these disposition parameters can be identified simultaneously. FcRn-mediated linear clearance and central volume of distribution generally scale reliably from non-human primate data and can be estimated with reasonable confidence, whereas TMDD parameters such as target baseline concentration, target turnover, and binding constants are often poorly identifiable from preclinical pharmacokinetic data at this stage and are commonly fixed to in vitro or literature values while the linear disposition parameters are estimated, paralleling the structural and practical identifiability concerns raised for physiologically based models in Section 10.5 [50,51,52].

Eculizumab exemplifies a more extreme case. It binds human complement component C5 with high affinity but does not cross-react with rodent C5. Conventional rodent PK and pharmacology studies are therefore essentially uninformative regarding target engagement. Human dose selection relied on ex vivo human assays and PK/PD modelling anchored in non-human primate data, where cross-reactivity exists [53]. Bevacizumab and trastuzumab illustrate the more typical situation in which TMDD measurably shapes PK at therapeutically relevant doses, particularly in patients with high tumor burden or shed antigen, requiring two-compartment or full TMDD models rather than empirical allometric extrapolation alone [47,50]. The practical consequence for FIH dose selection is that MABEL-based dosing, anchored in human cell or tissue potency assays, generally outranks allometric MRSD estimates for biologics targeting novel pathways [47,54].

### 4.6. Intestinal Lymphatic Transport

For highly lipophilic drugs, a fraction of the absorbed dose can bypass the portal circulation entirely and reach the systemic circulation via the intestinal lymphatics, associated with triglyceride-rich lipoproteins (chylomicrons) assembled within the enterocyte [28,29]. This route becomes quantitatively relevant only for compounds that combine high lipophilicity with appreciable long-chain triglyceride solubility, for which a log P greater than 5 and a lipid solubility above approximately 50 mg/g are commonly cited as approximate thresholds [29,30]. Because lymphatic delivery reaches the systemic circulation while bypassing hepatic first-pass metabolism, it can materially increase the oral bioavailability of affected compounds and lower their apparent first-pass loss in a manner that a body-weight regression of systemic clearance or volume cannot anticipate [28,29,30].

The interspecies scaling behavior of this pathway is instructive and differs from that of clearance and volume of distribution. In a cross-species study spanning mice, rats, dogs, and lymph cannulated human surgical subjects, intestinal lymph flow rate and triglyceride mass transport scaled allometrically with body mass, with exponents of approximately 0.84 to 0.94 and 0.80 to 0.96, respectively, such that weight-normalized lymph flow and lipid transport were lower in larger species [31]. Lymphatic mass transport of the lipophilic model drug halofantrine, by contrast, increased in a greater-than-proportional manner with body mass, with an exponent of approximately 1.3, an effect attributed to increased partitioning of the drug into lymph rather than blood as body size increased [31]. The practical implication for first-in-human prediction is consequential: lymphatic drug transport in humans most closely resembles that of larger species such as the dog. Rodent studies systematically underestimate it, so a species panel weighted toward rodents may misrepresent the human lymphatic contribution [31]. Reassuringly, the same study demonstrated that human lymph flow and lipid transport could themselves be predicted from animal data by allometric scaling, indicating that the lymphatic route is tractable to scaling provided it is modelled explicitly rather than subsumed into a single systemic exponent [31].

Several additional features make lymphatic transport poorly suited to simple body weight allometry of plasma pharmacokinetics. The extent of transport is highly sensitive to prandial state and to the type and quantity of co-administered lipid, which differ across preclinical study designs and between the fasted conditions typical of first-in-human protocols and the fed conditions under which lymphatic transport is greatest [29,30]. The lymphatic route also exposes lymph-resident tissues, including the mesenteric lymph nodes and circulating lymphocytes, to drug concentrations far higher than those measured in systemic plasma, a property now exploited deliberately for lymph-targeted therapy but also a source of disconnect between plasma-based scaling and tissue exposure [32]. For lipophilic candidates, the lymphatic contribution is therefore best characterized directly in a lymph-cannulated model, ideally in a larger species, and incorporated explicitly into IVIVE or PBPK rather than inferred from a plasma concentration–time course alone [30,31,32].

### 4.7. Toxicodynamic Species Differences: A Counterpart Failure Mode

Allometric scaling addresses the question of how much drug reaches the systemic circulation across species, but not whether the pharmacological or toxicological consequences of that exposure are equivalent across species. Thalidomide is the historical paradigm. Marketed from 1957 as a sedative and antiemetic, it produced an estimated 10,000 cases of phocomelia and related limb-reduction defects before withdrawal in 1961. The catastrophe was not a failure of pharmacokinetic scaling. Rather, the rodent species used for the original toxicology programs were largely resistant to thalidomide teratogenicity, while rabbits and primates are sensitive. The molecular basis is now understood to involve binding to cereblon (CRBN), a substrate receptor for the CRL4 E3 ubiquitin ligase, leading to downstream degradation of developmentally critical transcription factors; species-specific cereblon structures and substrate handling explain much of the interspecies divergence [55]. The case directly drove the development of ICH S5 reproductive toxicology guidelines mandating evaluation in two species, typically one rodent and one non-rodent, and underlies the broader principle that species panels for toxicodynamic endpoints must be selected on mechanistic grounds rather than convention [55,56]. Thalidomide thus complements the pharmacokinetic case studies of Section 7: even a compound whose disposition scales adequately between species can still fail catastrophically in humans if the relevant toxicodynamic biology is not represented in the preclinical panel. The fenfluramine/dexfenfluramine combination, withdrawn in 1997 after reports of valvular heart disease, provides a more contemporary illustration of the same principle: rats and dogs do not develop the human-pattern valvulopathy on short-term dosing, and the underlying 5-HT_2_B receptor–driven myofibroblast proliferation on cardiac valves only declared itself with chronic human exposure [57]. Mechanism-anchored receptor screening (off-target 5-HT_2_B affinity) is now a routine pre-clinical filter for compounds with serotonergic or related cardiovascular liabilities, precisely because body-weight allometric scaling will not flag this risk. Where the breakdown is quantitative rather than categorical, however, several empirical corrections can partly rescue the body-weight relationship, which is the subject of the next section.

## 5. Correction Factors and the Rule of Exponents

Recognizing the limitations of simple allometry, various correction factors have been proposed to improve the accuracy of human clearance predictions [9,58,59,60]. The two most widely applied corrections involve maximum life-span potential (MLP) and brain weight (BrW).

### 5.1. Maximum Life-Span Potential and Brain Weight Corrections

Mahmood and Balian [5] demonstrated that plotting CL × MLP or CL × BrW against body weight can improve the predictive accuracy of allometric scaling for certain drugs. Mathematically, these corrections are equivalent to multiplying the predicted human clearance from simple allometry by a constant (F^MLP^ or F^B^_r_^W^), the value of which depends on the species combination used [58]. Tang and Mayersohn [58] showed that, across all species combinations studied, F^B^_r_^W^ is consistently larger than F^MLP^ by approximately 1.3–1.9, and that different species combinations yield different correction factor values, introducing an element of arbitrariness.

### 5.2. The Rule of Exponents

The rule of exponents (ROE), formalized by Mahmood and Balian [5], provides practical guidance for selecting the appropriate correction based on the allometric exponent observed from simple scaling. When the exponent falls between 0.55 and 0.70, simple allometry alone reasonably well predicts clearance. When the exponent lies between 0.71 and 1.0, plotting CL × MLP against body weight significantly improves predictions. When the exponent exceeds 1.0, a two-term power equation incorporating CL × BrW and body weight is recommended [6,9]. Tang and Mayersohn [59] noted an intrinsic limitation of this approach: different study designs (e.g., different species combinations, dose levels, or PK sampling schemes) can yield significantly different exponents and, consequently, different predictions for the same drug. Nevertheless, the ROE has demonstrated practical utility across numerous examples and remains a useful heuristic in the allometric toolkit [6,58,59]. Theophylline is the textbook case in which simple multi-species allometry yields a clearance prediction within a clinically tolerable margin of the observed human value. Its long pre-IVIVE history of cross-species dose management by allometric reasoning reflects this favourable behaviour [61]: principally cleared by hepatic CYP1A2, exhibiting modest interspecies variability, theophylline falls comfortably within the 0.55–0.70 exponent range where simple allometry performs adequately under ROE guidance. In contrast, contemporary pharmacometric analyses have questioned the reproducibility and mechanistic interpretability of ROE-based corrections across chemically diverse datasets [6,21,59]. The exponent assigned to a given compound can shift between cut-off bands when the species panel is changed or when a single outlier is added or removed, and the choice between MLP and BrW corrections lacks a deep biophysical basis. Modern practice, therefore, treats the ROE as a heuristic before being cross-checked against IVIVE and PBPK rather than as a standalone decision rule.

### 5.3. Single-Species Scaling with Liver Blood Flow Correction

An alternative approach, particularly useful early in development when data from only one preclinical species may be available, involves single-species allometric scaling with liver blood flow (LBF) correction [23,60,62]. Caldwell et al. [62] examined whether human clearance, volume of distribution, and half-life can be predicted solely from in vivo rat data. They reported that single-species scaling, particularly when anchored to physiologically based corrections, can yield acceptable predictions for many small molecules. Nagilla and Ward [60] demonstrated that scaling from rat or monkey using LBF-adjusted clearance can yield predictions comparable to those from multi-species allometry for drugs primarily cleared by hepatic metabolism. This approach explicitly accounts for the physiological constraint that hepatic clearance cannot exceed liver blood flow, reducing the risk of gross overestimation. Miller et al. [23] further showed that integrating such single-species clearance predictions with PBPK models can enhance FIH prediction accuracy for challenging compounds. These corrections sharpen point estimates of clearance and volume; recovering the full human concentration–time profile, rather than isolated parameters, calls for the profile-superposition approach described next.

## 6. Predicting Concentration–Time Profiles: The Wajima Normalization Method

While the preceding sections focus on predicting individual PK parameters (CL, V_D_, t½), the complete concentration–time profile is often more clinically useful for dose selection and safety assessment. Wajima et al. [63] developed a method for predicting human plasma concentration–time profiles from animal data, building on the earlier Dedrick plot approach [13] but with enhanced flexibility.

### 6.1. Normalization Principles

The Wajima method assumes that concentration–time profiles are similar among species when appropriately normalized. The normalized time (t′) is obtained by dividing time (t) by the mean residence time (MRT = V_ss_/CL), whereas the normalized concentration (C′) is obtained by dividing the plasma concentration (C) by the ratio of dose to steady-state volume of distribution (Dose/V_ss_). The resulting dimensionless curves from different species should approximately superimpose if the assumption holds [63]. This is mathematically expressed as:

*t*′ = *t*/*MRT*; C′ = *C*/(*Dose*/*V_ss_*) (2)

For a one-compartment intravenous bolus model, the normalized curve simplifies to C′(t′) = e^−t^′, which is species-independent. More complex multi-compartmental profiles yield normalized curves of the form C′(t′) = A e^−^α^t^′ + B e^−^β^t^′, where A, B, α, and β are fitted from pooled normalized data [63].

### 6.2. Operational Workflow

The complete workflow proceeds as follows: (1) measure or estimate CL, V_ss_, and dose in preclinical species; (2) compute species-specific MRT and Dose/V_ss_; (3) normalize the time and concentration axes to build the dimensionless profile; (4) predict human CL and V_ss_ using any appropriate method (allometry, IVIVE, PBPK); (5) de-normalize the composite curve using predicted human MRT and Dose/V_ss_ to generate the human concentration–time profile [63]. The critical advantage of this approach is its flexibility: it can use CL and V_ss_ predictions from any method, not just allometry.

**Figure 3:**
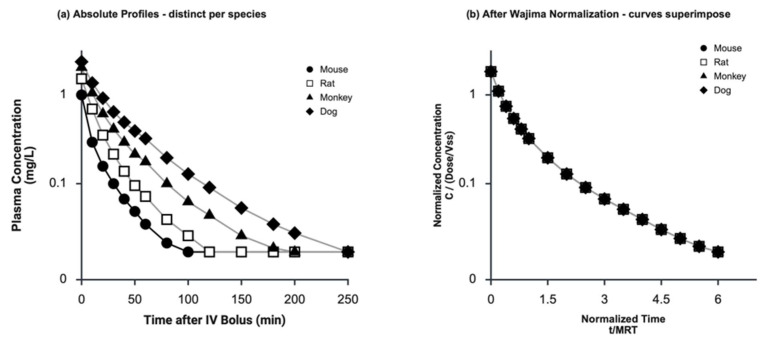
Wajima multi-species worked example after a 1 mg/kg IV bolus in mouse, rat, monkey, and dog. (**a**) Absolute plasma concentration–time profiles, plotted on a log *y*-axis, differ markedly across species in both timescale and concentration range. (**b**) After Wajima normalization (*x*-axis rescaled to t/MRT, *y*-axis rescaled to C/(Dose/V_ss_)), the four profiles collapse onto a single common curve, demonstrating the superposition principle on which forward projection to humans relies. Parameter values and exposure-metric dispersion are tabulated in Appendix A (Table A4 and Table A5).

To illustrate the method, consider a hypothetical small molecule administered intravenously to four preclinical species at the same body-weight-normalized dose. The species-specific parameters (CL, V_ss_, MRT) might be: mouse (CL = 2.25 mL/min, V_ss_ = 37.5 mL, MRT ≈ 16.7 min); rat (CL = 12.5 mL/min, V_ss_ = 250 mL, MRT ≈ 20 min); monkey (CL = 125 mL/min, V_ss_ = 4 L, MRT ≈ 32 min); dog (CL = 180 mL/min, V_ss_ = 7 L, MRT ≈ 38.9 min). Plotted on absolute axes, the four species concentration–time curves differ markedly in both peak height and duration. When time is rescaled to t/MRT and concentration to C/(Dose/V_ss_), the four curves substantially superimpose, supporting use of the composite profile as the kernel from which the human concentration–time course is recovered by de-normalization with the predicted human CL and V_ss_ [63]. These absolute and rescaled profiles are shown in Figure 3, where the species-specific curves separate on absolute axes (Figure 3a) and collapse onto a single common curve after Wajima normalization (Figure 3b).

### 6.3. Assumptions and Limitations

The Wajima method assumes linear (dose-proportional) pharmacokinetics and shape similarity of normalized profiles across species. When metabolism is saturable, clearance mechanisms are species-specific (e.g., active transport, unique enzyme systems), or extensive first-pass metabolism introduces non-linearity, the normalized curves may not superimpose, and predictions can be unreliable [63,64]. Phenytoin is the canonical example: its clearance is governed by capacity-limited (Michaelis–Menten) CYP2C9 and CYP2C19 metabolism such that small dose increases at therapeutic levels can produce disproportionately large rises in steady-state concentration [65]. Neither simple allometric scaling nor Wajima normalization captures this non-linearity, and human dose individualization requires explicit Michaelis–Menten parameterization derived from patient-level data. Protein binding differences (f_u_), blood/plasma partitioning ratios, and active transport mechanisms represent additional confounders not captured by the simple normalization. Using multiple species to build the composite normalized curve improves robustness, and the quality of the human prediction depends primarily on the accuracy of the CL and V_ss_ predictions [18,63].

## 7. Case Vignettes That Rewrote the Map

Some of the most consequential failures (Table 3) in translational pharmacology arose from human-specific liabilities that no plausible allometric scaling could have anticipated, fundamentally reshaping FIH doctrine and regulatory expectation [66,67,68]. These cases underscore that allometric scaling, while useful for initial parameter estimation, cannot substitute for a mechanistic understanding of species-specific biology.

To read these cases as a structured map rather than a catalog, it helps to group them by the failure mode each exemplifies. Benoxaprofen (Section 7.8) is the clearance-scaling failure proper: human elimination proved far slower than body-weight projection implied, and the drug accumulated dangerously in elderly patients. Cerivastatin (Section 7.7) and terfenadine (Section 7.9) are metabolism- and interaction-dependent failures, in which a cytochrome P450 drug interaction rather than body size dictated the clinically decisive exposure. TGN1412 (Section 7.2) is a target- and immune-mediated failure, in which receptor biology rather than pharmacokinetics governed the outcome and which only MABEL-anchored dosing could have contained. Fialuridine (Section 7.1) and troglitazone (Section 7.4) are intrinsic toxicodynamic failures, driven by human-relevant mitochondrial injury and reactive-metabolite formation, respectively. In contrast, BIA 10-2474 (Section 7.3) and torcetrapib (Section 7.5) are off-target toxicities that surfaced only at clinical exposures. Rofecoxib (Section 7.6) is an on-target, mechanism-based class effect. Across all of these modes, the lesson is the same: interspecies pharmacokinetic scaling is necessary but not sufficient, and the decisive liability often lies in biology that body weight does not index.

### 7.1. Fialuridine (FIAU)

Fialuridine, an antiviral nucleoside analogue, caused catastrophic hepatic failure and lactic acidosis in a 1993 clinical trial, resulting in five deaths and two liver transplantations. Preclinical studies across multiple species showed no indication of the severe mitochondrial toxicity observed in humans, because fialuridine’s incorporation into mitochondrial DNA is mediated by a human-specific mitochondrial transporter [69,70]. This case established the principle that human-relevant assays and human-specific toxicity screening are essential complements to allometric dose selection.

### 7.2. TGN1412 (CD28 Superagonist)

The 2006 TGN1412 phase I trial produced life-threatening cytokine storm in all six healthy volunteers at a dose 500-fold below the no-observed-adverse-effect level (NOAEL) in cynomolgus monkeys. The failure stemmed from fundamental immunological differences: the distribution of the CD28 receptor on effector memory T cells in humans versus non-human primates produced a qualitatively different pharmacological response [54,71,72]. This catastrophe catalyzed the European Medicines Agency’s revised guideline mandating MABEL-based dosing for high-risk biologics, ex vivo human tissue assays, and conservative sentinel-dosing designs [68].

### 7.3. BIA 10-2474 (FAAH Inhibitor)

In 2016, the fatty acid amide hydrolase (FAAH) inhibitor BIA 10-2474 caused fatal neurological injury in one volunteer and severe brain damage in others during a multiple-ascending-dose study. Preclinical species showed adequate tolerability, but off-target effects on lipases beyond FAAH led to CNS toxicity unique to humans at the administered doses [73,74]. The incident underscored the need for slower dose escalation, real-time biomarker monitoring, and neuroimaging surveillance in early clinical trials of CNS-active compounds.

### 7.4. Troglitazone

Troglitazone, the first marketed thiazolidinedione, was withdrawn after reports of severe idiosyncratic hepatotoxicity, including fulminant liver failure and transplantation. Mechanistic studies subsequently identified bioactivation by CYP3A4 and CYP2C8 to reactive quinone-like metabolites and ring-opened sulfenic acid intermediates that covalently bind cellular macromolecules and trigger mitochondrial injury, neither of which was reliably flagged by standard preclinical hepatotoxicity assays [75,76]. The case led to enhanced drug-induced liver injury (DILI) screening paradigms, including reactive-metabolite trapping, mitochondrial function assays, and signal-detection strategies based on covalent binding burden per daily dose.

### 7.5. Torcetrapib

Torcetrapib, a cholesteryl ester transfer protein (CETP) inhibitor designed to raise HDL-cholesterol, produced favourable surrogate-biomarker changes in trials but increased all-cause mortality in the ILLUMINATE outcome study. The mortality signal traced not to CETP inhibition itself but to off-target hemodynamic effects, including aldosterone elevation, blood pressure increases, and electrolyte shifts independent of the lipid mechanism [25]. The case reinforced the imperative to interrogate mechanisms beyond surrogate endpoints and to incorporate vascular and electrolyte pharmacology into the safety package of compounds intended for long-term cardiovascular use.

### 7.6. Rofecoxib

Rofecoxib, a selective COX-2 inhibitor, was withdrawn from the market in 2004 following the VIGOR and APPROVe trials, which revealed a sustained excess of thrombotic cardiovascular events. The mechanism is generally ascribed to disruption of the prostacyclin–thromboxane balance, with COX-2-dependent vascular prostacyclin suppressed, without a compensatory effect on platelet COX-1-dependent thromboxane A_2_ production [77,78]. Animal efficacy and short-term human safety data had been reassuring; only long-term cardiovascular outcome trials surfaced the signal. The case crystallized the need for cardiovascular outcome trials for anti-inflammatory agents and broader chronic-use compounds, where short-term biomarker improvements may mask longer-term harm.

### 7.7. Cerivastatin

Cerivastatin, withdrawn worldwide in 2001 because of a disproportionate rate of fatal rhabdomyolysis, illustrates how a pharmacokinetic interaction that preclinical species do not flag can dominate the clinical safety profile. The rhabdomyolysis risk was concentrated in patients co-prescribed gemfibrozil, an inhibitor of both CYP2C8-mediated cerivastatin metabolism and OATP1B1-mediated hepatic uptake, resulting in several-fold increases in exposure relative to monotherapy [79]. Neither standard CYP3A4-centric DDI screening (cerivastatin is principally a CYP2C8 substrate) nor allometric extrapolation captured this liability. The case is now a canonical example of why ECCS classification, transporter-DDI screening, and post-marketing pharmacoepidemiology must be integrated alongside any allometric or PBPK extrapolation [41,43,44,79].

### 7.8. Benoxaprofen

Benoxaprofen, a propionic acid NSAID introduced in 1980, was withdrawn worldwide in 1982 following reports of hepatotoxicity and severe photosensitivity, with elderly patients disproportionately represented among fatalities. The pharmacokinetic profile illustrates a textbook failure mode of body-weight allometric scaling. In rats and dogs, the elimination half-life was on the order of single-digit hours, whereas in healthy young adult human volunteers, the half-life extended to approximately 20–30 h, a divergence that simple multi-species body-weight scaling could not have anticipated [80]. The principal clearance pathway is acyl glucuronidation followed by renal excretion of conjugates, and species differences in UGT-mediated conjugation efficiency, combined with the absence of an analogous renal-handling step in rodents, led to the disproportionate extension of the human half-life [80]. In elderly subjects with age-related decline in glomerular filtration, the half-life lengthened further to 100 h or more, and steady-state concentrations of the parent drug and the reactive acyl glucuronide accumulated to several-fold higher levels than in younger adults [81]. The exposure surface that drove hepatic and cutaneous toxicity was therefore a subpopulation phenomenon largely invisible to a development program anchored on rodents, dogs, and young adult human volunteers. The lessons closely parallel those of cerivastatin: subpopulation-amplified pharmacokinetic deviations require pre-marketing exposure characterization in elderly and renally impaired cohorts, and reliance on simple allometric scaling for compounds with slow human glucuronidation or renal-amplification pathways is insufficient [80,81].

### 7.9. Terfenadine

Terfenadine, a non-sedating H_1_ antihistamine introduced in the 1980s and withdrawn in 1998, illustrates a distinct failure mode: the parent compound is a potent hERG channel blocker but is normally clinically silent because of extensive first-pass metabolism by CYP3A4 to the active metabolite fexofenadine. Reports of torsades de pointes and sudden death emerged when terfenadine was co-administered with CYP3A4 inhibitors such as ketoconazole, erythromycin, and the constituents of grapefruit juice, conditions under which parent terfenadine accumulated to concentrations sufficient to prolong QTc and trigger ventricular arrhythmia [82,83]. The pharmacokinetic structure of the failure is instructive. Body-weight allometric scaling applied to total drug exposure would not have flagged a problem, because in monotherapy, preclinical species and humans both metabolize terfenadine efficiently, and parent plasma concentrations remain low. The risk surface was hidden in three interacting features that allometry alone cannot interrogate: (i) species differences in CYP3A subfamily activity and substrate specificity that bear on the predictability of fractional first-pass metabolism in humans; (ii) the inversion of the parent-to-metabolite exposure ratio under CYP3A4 inhibition, converting a notionally safe prodrug system into a hERG-blockade liability; and (iii) the absence of a routine preclinical assay for QT prolongation before ICH E14 and ICH S7B [82,83]. The case directly catalyzed contemporary requirements for thorough QT studies and hERG screening, the metabolite-aware DDI evaluations that ECCS classification now anchors [41,43,44], and the broader regulatory expectation that compounds with active metabolites or saturable first-pass pathways be characterized across realistic co-medication scenarios rather than in isolation. Terfenadine is also the canonical example that PBPK platforms now explicitly address through metabolite-linked parent models and DDI simulations [22,84,85]. These vignettes are summarised collectively in Table 4.

### 7.10. Successful Allometric Predictions

The preceding vignettes emphasize failure modes, but allometry earns its place in early development because it also works, often impressively, for compounds whose disposition is governed by size-dependent physiology rather than by species-specific biochemistry. Theophylline is a classic teaching example: its clearance and volume of distribution scale predictably across species, so body-weight-based projection provides a serviceable first estimate of human exposure [61]. More generally, drugs cleared predominantly by glomerular filtration or by physiological processes conserved across mammals tend to scale well; renally eliminated small molecules such as the aminoglycoside gentamicin fall into this category, and systematic comparisons confirm that simple allometry and its refinements predict human clearance most reliably for this class [18,26]. Therapeutic proteins provide the most striking modern success. Because the disposition of monoclonal antibodies is dominated by largely conserved catabolic and FcRn-mediated processes rather than by divergent metabolic enzymes, scaling from non-human primates predicts human clearance and volume with an accuracy that small molecules rarely achieve [26,47]. These successes are not in tension with the failure cases; together they define the operating envelope of allometry, which performs best when elimination is flow- or filtration-limited and conserved across species, and worst when it hinges on species-divergent enzymes or transporters.

## 8. From NOAEL to a Conservative First Step

Translating preclinical toxicology findings into a defensible FIH starting dose proceeds along complementary routes that should be reconciled before exposing human volunteers [15,67,86].

### 8.1. The Body Surface Area (BSA) Approach

The dose-by-factor pathway converts the animal no-observed-adverse-effect level (NOAEL) to a human equivalent dose (HED) using fixed body surface area (BSA) K_m_ conversion factors. The HED is calculated as NOAEL × (K_m_, animal/K_m_, human), where K_m_ is the ratio of body weight to body surface area for each species. A safety factor (typically 10-fold) is then applied to obtain the MRSD [15,67]. Table 5 illustrates this conversion across common preclinical species.

### 8.2. Triangulation with MABEL and PBPK

For high-risk compounds, particularly biologics or those acting on novel targets, MABEL-based dosing provides a complementary pharmacological anchor [54,68]. MABEL is derived from in vitro potency data (e.g., EC_10_ from receptor-binding or functional assays) and ex vivo human tissue responses. It identifies the lowest dose at which a biological effect is anticipated. PBPK model qualification and transparent reporting [22] help reconcile BSA-scaled MRSD with mechanism-anchored MABEL, ensuring that the more conservative estimate governs FIH initiation. The FDA [67], EMA [68], and ICH M3(R2) [66] guidelines collectively endorse this weight-of-evidence approach.

## 9. Accounting for Variability and Special Populations

Generalization beyond healthy adults requires explicit consideration of physiological diversity that simple allometric scaling cannot capture [22,87,88,89]. Table 6 provides a planning matrix to anticipate shifts in exposure across key special populations and clinical scenarios.

Pediatric dosing should incorporate enzyme and transporter ontogeny rather than rely on simple weight- or BSA-based exponents [88,89]. CYP3A4, for example, matures over the first year of life, while CYP1A2 and CYP2D6 exhibit distinct developmental trajectories. Population PBPK models that embed age-dependent physiology (organ volumes, blood flows, enzyme expression, and renal function) can simulate pediatric exposures and justify bridging strategies based on adult data [22,88,89]. Similarly, renal and hepatic impairment alter intrinsic clearance, organ perfusion, and protein binding, requiring explicit physiological modelling rather than simplistic scaling [42,90]. Two clinically familiar examples anchor the point: metformin is almost entirely cleared unchanged by glomerular filtration and active tubular secretion via OCT2 and MATE transporters, with the result that even modest declines in eGFR translate into exposure increases that allometric body-weight scaling alone would miss, hence the eGFR-based contraindications and dose adjustments in current labelling [46,90]. Vancomycin, similarly renally cleared, requires therapeutic drug monitoring in patients with even mild renal impairment because of its narrow therapeutic index, and consensus guidelines are anchored in eGFR-based dose bands rather than allometric extrapolation from preclinical species [90].

## 10. Platforms That Operationalize Allometry Within PBPK

Contemporary PBPK platforms enable development teams to treat allometric scaling as a testable hypothesis embedded within mechanistic models, rather than as a standalone prediction tool [12,22,91]. The three principal commercially available platforms each offer distinct strengths.

### 10.1. Simcyp^®^

Simcyp^®^ Simulator Version 24 (Certara) provides curated virtual populations spanning diverse demographics and disease states, with detailed CYP and transporter ontogeny modules. The platform incorporates the Advanced Dissolution, Absorption and Metabolism (ADAM) model for mechanistic oral absorption simulation alongside its long-established disposition framework, complementing its strengths in CYP- and transporter-mediated DDI prediction and pediatric extrapolation [22,92]. Simcyp^®^ has become the most widely used platform for regulatory PBPK submissions, and its population-based approach generates predicted exposure distributions rather than single-point estimates, enabling risk-based decision-making.

### 10.2. GastroPlus^®^

GastroPlus^®^ X (GPX) (Simulations Plus) integrates the Advanced Compartmental Absorption and Transit (ACAT) model with PBPK disposition, with modules for modelling oral absorption and formulation effects. Current releases also include a dedicated drug–drug interaction (DDI) module and validated special-population physiologies (pediatric, geriatric, renal- and hepatic-impaired, pregnant), providing capabilities aligned with those of Simcyp^®^. The integration of allometric scaling with mechanistic absorption, DDI, and elimination modules makes GastroPlus^®^ applicable to FIH dose selection of orally administered drugs and for label-supportive simulations in special populations [93].

### 10.3. PK-Sim^®^/MoBi^®^

PK-Sim^®^/MoBi^®^ (Open Systems Pharmacology Suite Version 12) provides an open-source platform with organ-resolved anatomy, enzyme/transporter ontogeny, and flexible model building through MoBi^®^’s graphical interface. Its transparency and extensibility have made it increasingly popular in academic and regulatory settings [94].

### 10.4. Phoenix WinNonlin^®^

Phoenix WinNonlin^®^ Version 8.7 (Certara) remains foundational for non-compartmental and population PK analyses and is often used to compute empirical allometric fits from preclinical data before export to PBPK platforms. Its allometric module automates the regression and correction-factor selection described in Section 2 and Section 5 [6].

The principal software platforms used for PBPK and pharmacokinetic modeling, together with their primary scope, typical context of use, and representative evidentiary expectations, are summarized in Table 7.

### 10.5. Regulatory Acceptance

Across recent FDA submissions, PBPK analyses, especially for CYP-mediated DDIs, are increasingly accepted when platform qualification and drug-model verification are documented transparently [22,85,87]. Regulatory acceptance varies by intended impact: supportive PBPK contextualizing other data is widely accepted; decision-enabling PBPK that drives dose recommendations or DDI labelling demands closer scrutiny; and label-replacing PBPK substituting for dedicated clinical studies remains the most stringently reviewed category, restricted to thoroughly verified contexts of use [22,84,85]. This graded expectation is reflected in the FDA and EMA guidances on PBPK analyses and reporting, under which the depth of platform qualification and model verification expected scales with the regulatory impact of the question addressed [95,96]. Sun et al. [85] reported that, between 2019 and 2023, PBPK modelling supported regulatory decisions in the majority of novel drug approvals. PBPK is not without its own failure modes: parameter identifiability can be poor when many tissue partition coefficients and enzyme abundances are simultaneously fitted from limited data; uncertainty propagation through complex models can produce confidence intervals that under-represent true predictive uncertainty; overparameterization may yield apparent good fits without genuine predictive validity; transporter abundance values carry substantial measurement uncertainty across donors and tissues; and predictivity for poorly soluble, formulation-sensitive compounds depends heavily on the quality of dissolution and precipitation modelling inputs [12,84,91]. The integration of allometric priors with PBPK mechanistic refinement represents the current state of the art for interspecies translation, provided that these limitations are acknowledged and addressed during model qualification [12,23,84].

## 11. The Extended Clearance Classification System

Varma et al. [41] proposed the Extended Clearance Classification System (ECCS), which categorizes drugs based on their molecular properties (molecular weight, ionization state, and permeability) to predict the dominant clearance mechanism. ECCS builds on the foundational Biopharmaceutics Drug Disposition Classification System (BDDCS) framework articulated by Wu and Benet, which first formalized the interplay between permeability, solubility, transporters, and metabolism in shaping disposition [45]. ECCS provides a priori insight into whether a compound’s clearance is likely dominated by CYP-mediated metabolism (where allometric scaling with IVIVE tends to perform well), renal filtration (where GFR-based scaling is appropriate), or transporter-mediated hepatic uptake or biliary excretion (where simple allometry may fail). The framework thus helps practitioners decide, before data are generated, which scaling approach is most likely to yield reliable human predictions and where additional mechanistic studies may be needed [41,42,45].

## 12. Artificial Intelligence and Machine Learning in Allometric and PBPK Prediction

The past five years have seen rapid uptake of machine learning (ML) and broader artificial intelligence (AI) methods alongside, and increasingly within, traditional pharmacokinetic modelling [97,98]. Three application classes are now evident in the FIH literature. First, ML-assisted parameter optimization: gradient-boosted tree models, neural networks, and Gaussian process regressions have been deployed to predict in vitro intrinsic clearance, fraction unbound, blood-to-plasma ratio, and other ADME parameters from molecular descriptors, generating priors that feed directly into allometric and PBPK workflows when measured values are unavailable [98]. Second, Bayesian updating and hybrid PBPK–ML frameworks: PBPK models can be embedded in a Bayesian framework that updates posterior distributions for tissue partition coefficients, enzyme abundances, and transporter activities as preclinical and early human data accumulate, with ML emulators substituting for full PBPK simulation during repeated sampling [84,98]. Third, virtual-population generation and AI-guided DDI prediction: generative models can construct virtual cohorts that span realistic correlations among demographics, genotype, and physiology, while supervised classifiers screen large compound libraries for likely victim or perpetrator behaviour in CYP- or transporter-mediated DDIs [97,98].

For interspecies translation specifically, ML approaches do not replace allometric or PBPK reasoning but rather complement it. They are most useful when mechanistic priors are weak (e.g., early discovery, novel chemotypes) and when large historical datasets are available to train the relevant model. Their principal risks are familiar from other ML domains: poor extrapolation outside the training distribution, opacity of feature importance, and over-reliance on retrospective benchmarks that may not reflect the prospective FIH setting [98]. Hybrid model-informed drug development frameworks, in which mechanistic PBPK models retain interpretability while ML emulators accelerate computation and ML priors fill gaps in measured parameters, represent the most defensible path forward for regulatory submissions [22,84,97,98]. This hybrid posture is consistent with the foundational vision of pharmacokinetic–pharmacodynamic modelling articulated by Sheiner and Steimer [99], in which mechanistic models, sparse data, and Bayesian updating combine to inform dose selection across the development lifecycle.

## 13. A Decision Path: Integrated Framework for PK and Dose Predictions

The weight of evidence supports a disciplined, mechanism-anchored approach to interspecies extrapolation that integrates multiple methodologies rather than relying on a single technique [6,22,23,86]. This review proposes the following stepwise framework.

Step 1: Species selection and qualification. Before any extrapolation, assess whether the available preclinical species are mechanistically relevant to the anticipated human disposition and pharmacology, and exclude those that are not. The aim is to confirm that the enzymes, transporters, plasma protein binding, and pharmacological target that govern the compound in humans are adequately represented in at least one species in the panel, and to recognize where they are not (for example, the absence of N-acetyltransferase 2 in the dog, rat-specific CYP isoforms, species lacking cross-reactivity for a biologic target, or rodents resistant to a relevant toxicodynamic effect). Species that diverge mechanistically should be excluded rather than allowed to rotate the regression, and the rationale for inclusion or exclusion should be documented. This qualification step also determines whether the panel can support a body-weight regression at all, or whether mechanism-specific or human-relevant in vitro approaches must carry the prediction [6,18,21].

Step 2: Empirical allometry with ECCS classification. Classify the compound under the ECCS to identify the dominant anticipated clearance mechanism (CYP-mediated metabolism, renal filtration, or transporter-mediated hepatic uptake or biliary excretion) [42]. Because the ECCS, and the related BDDCS, classify a compound from its physicochemical and in vitro properties (molecular weight, ionization, and passive permeability for the ECCS; solubility, permeability, and the extent of metabolism for the BDDCS), the class can be assigned before any human data are generated and is therefore available at the first-in-human stage [41,45]. Whether nonlinear pharmacokinetics should be anticipated is likewise judged before human dosing, from preclinical dose-ranging studies that reveal dose-disproportional exposure or saturation, from in vitro evidence that metabolizing enzymes or transporters approach saturation at projected clinical concentrations, and, for biologics, from target expression and binding kinetics that predict target-mediated drug disposition [41,50,65]. For biologics in particular, this also recognizes that not all parameters are simultaneously identifiable: FcRn-mediated linear clearance and central volume can be carried from non-human primate data, whereas poorly identifiable TMDD parameters are fixed to in vitro or literature values rather than estimated [50,51] (Section 4.5). This classification informs whether simple allometric scaling is likely to be reliable or whether mechanism-specific scaling (e.g., GFR-based scaling, transporter-aware IVIVE) should be prioritized. Begin with simple allometric scaling across available preclinical species to frame plausible bounds for CL, V_D_, and t½. Evaluate the allometric exponent against the rule of exponents and apply correction factors where indicated [5,6,58].

Step 3: Mechanistic IVIVE. Integrate in vitro metabolism and transporter data via the well-stirred or parallel-tube liver model to generate IVIVE-based clearance estimates. Compare with allometric predictions and investigate discrepancies [14,42,100,101].

Step 4: PBPK integration. Build a compound-specific PBPK model incorporating allometric priors, IVIVE-derived parameters, and measured physicochemical properties. Use the model to simulate expected human exposures and to test sensitivity to key assumptions and uncertainties [12,22,23,91].

Step 5: Wajima profile prediction. Where full concentration–time profiles from preclinical species are available, apply the Wajima normalized time-course method to predict the human profile shape, and cross-check against PBPK simulations [63]. When the four methods (empirical allometry, IVIVE, PBPK, and Wajima) yield human CL or exposure estimates that agree within approximately 2-fold, the convergence supports a single best estimate for dose selection. The approximately 2-fold window is the conventional acceptance criterion for human pharmacokinetic prediction rather than an arbitrary cut-off: retrospective evaluations of clearance and volume predictions report that the better allometric and IVIVE methods typically recover observed human values to within about 2-fold. PBPK verification practice has adopted the same bound, so agreement at this level reflects the practical resolution of the methods rather than genuine mechanistic identity [18,64,100,102,103]. Where they disagree by more than two-fold, the modeller should not average the discrepant estimates. The discrepancy itself should instead be treated as a diagnostic signal. Re-examine the ECCS classification and the dominant clearance assumption. Verify the IVIVE inputs (intrinsic clearance, fraction unbound in plasma and microsomes, blood-to-plasma ratio, and any transporter kinetics) using additional in vitro experiments where feasible. Check the PBPK model for sensitivity to tissue partition coefficients and enzyme abundance. Confirm that the Wajima profiles superimpose adequately across species. If the discrepancy cannot be reconciled mechanistically, the most conservative defensible estimate (typically the one predicting the highest human exposure or lowest clearance) should govern the FIH starting dose, with the rationale documented transparently for regulatory review [22,23,26].

Step 6: Dose selection triangulation. Triangulate the MRSD from BSA-based scaling with MABEL-derived starting doses and PBPK-simulated exposures. The most conservative estimate should govern FIH initiation, particularly for novel targets and biologics [15,66,67,68,86].

Step 7: Variability and uncertainty. Stress-test predictions via population PBPK simulations spanning anticipated special populations (pediatrics, renal/hepatic impairment, DDI scenarios). Iteratively update models with emerging human data [22,85,87].

**Figure 4:**
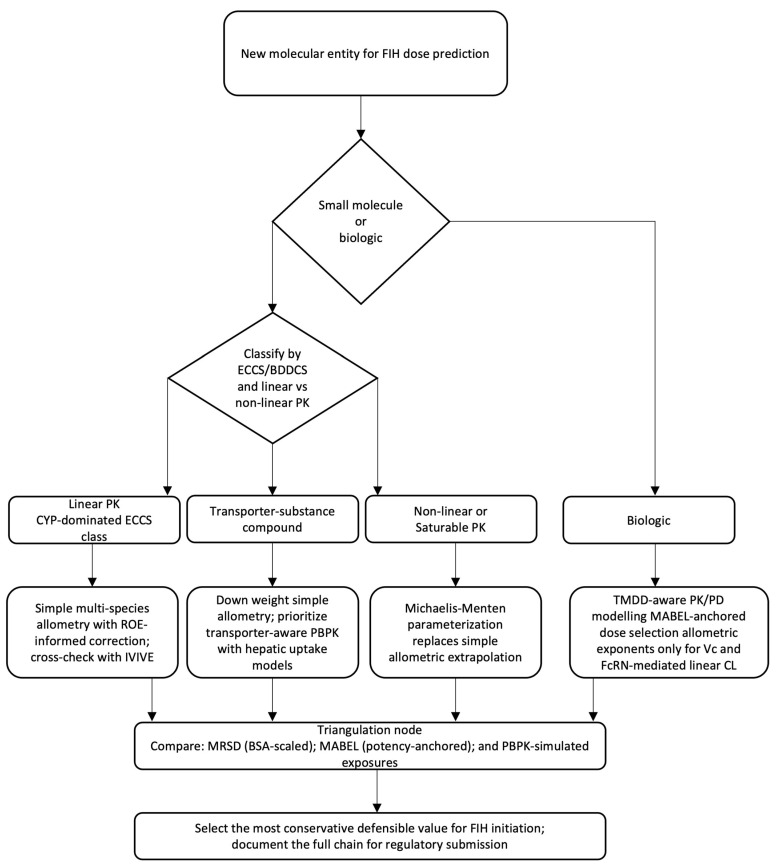
Practical decision tree for FIH human PK prediction. The algorithm begins by partitioning the molecule into small molecules versus biologics, then queries the ECCS/BDDCS class and whether linear or nonlinear pharmacokinetics are anticipated. For linear small molecules within CYP-dominated ECCS classes, simple multi-species allometry with ROE-informed correction provides the empirical starting point, cross-checked against IVIVE. For transporter-substrate compounds, simple allometry is downweighted and transporter-aware PBPK with explicit hepatic uptake modules is prioritized. For non-linear or saturable PK, Michaelis–Menten parameterization replaces simple allometric extrapolation. For biologics, TMDD-aware PK/PD modelling and MABEL-anchored dose selection govern, with allometric exponents used only to bound central-compartment volume and FcRn-mediated linear clearance empirically. All four branches converge at a triangulation node, where MRSD (BSA-scaled), MABEL (potency-anchored), and PBPK-simulated exposures are compared, with the most conservative, defensible value selected for FIH initiation and the full chain documented for regulatory submission. The tree is intended as a teaching aid and pre-data planning instrument; mechanistic findings during model qualification may legitimately override its default branches. Abbreviations: FIH, first-in-human; ECCS, Extended Clearance Classification System; BDDCS, Biopharmaceutics Drug Disposition Classification System; PK, pharmacokinetic; CYP, cytochrome P450; ROE, rule of exponents; IVIVE, in vitro-in vivo extrapolation; PBPK, physiologically based pharmacokinetic; TMDD, target-mediated drug disposition; PK/PD, pharmacokinetic/pharmacodynamic; MABEL, minimum anticipated biological effect level; Vc, central volume of distribution; FcRn, neonatal Fc receptor; CL, clearance; MRSD, maximum recommended starting dose; BSA, body surface area.

This framework treats allometry as a valuable starting hypothesis, one among several that must withstand scrutiny by mechanism and evidence, while anchoring the ultimate dose selection in the most robust available data [6,22,23,86]. The overall workflow is summarized in Figure 4.

Table 8 summarizes the recommended translational strategy, key limitations, and preferred complementary methods for common drug development scenarios.

**Table 8:** Best-practices checklist for selecting interspecies extrapolation methods by compound class and clinical scenario.

Situation	Recommended Approach	Avoid Relying Solely on	Preferred Supplementary Methods
Small molecules, CYP-dominated clearance	Multi-species allometry with ROE; IVIVE; PBPK verification	Simple allometry alone; rat-only scaling	Wajima profile prediction; LBF-adjusted single-species [5,14,23,60,62,63]
Transporter substrates (OATP, P-gp, BCRP, OCT/MATE)	Transporter-aware PBPK with explicit uptake/efflux kinetics	Total-CL allometry; CYP-only IVIVE	ECCS classification; sandwich hepatocyte uptake assays; pharmacogenetic stratification [41,42,43,44,45]
Biologics (mAbs and large proteins)	TMDD-aware PK/PD modelling; MABEL dose anchor	Small-molecule allometric exponents; NOAEL-only MRSD	Ex vivo human assays; species-cross-reactivity checks; sentinel dosing [47,50,54,68,71]
Pediatric extrapolation	Pediatric PBPK with enzyme/transporter ontogeny	BSA or body-weight scaling alone	Maturation functions; adult-to-pediatric bridging studies [22,87,88,89]
CNS-active compounds	PBPK with brain compartment; MABEL; slow MAD escalation	NOAEL-only MRSD; rapid escalation schedules	Off-target lipase/kinase screens; neuroimaging surveillance [68,73,74]
Non-linear or saturable PK	Michaelis–Menten parameterization; mechanistic PBPK	Linear PK assumptions; Wajima normalization	Repeat-dose PK with full Vmax/Km characterization; TDM [63,65]
Highly protein-bound drugs (>99% bound)	Unbound-CL allometry; PBPK with species-specific fu	Total-concentration allometry; cross-species fu pooling	Equilibrium dialysis at clinical concentrations; AAG measurement [19,20,35]

Note: The checklist is intended to support pre-data planning and reviewer-team discussions, not to substitute for compound-specific qualification. Mechanistic findings should override default branches whenever the empirical evidence warrants. The simple checklist above is expanded into the operational decision matrix below (Table 9), which additionally specifies the required supporting data, key diagnostic checks, and major sources of uncertainty for each compound class. Abbreviations: CYP, cytochrome P450; ROE, rule of exponents; IVIVE, in vitro-in vivo extrapolation; PBPK, physiologically based pharmacokinetic; LBF, liver blood flow; OATP, organic anion transporting polypeptide; P-gp, P-glycoprotein; BCRP, breast cancer resistance protein; OCT, organic cation transporter; MATE, multidrug and toxin extrusion transporter; ECCS, Extended Clearance Classification System; mAbs, monoclonal antibodies; TMDD, target-mediated drug disposition; PK/PD, pharmacokinetic/pharmacodynamic; MABEL, minimum anticipated biological effect level; NOAEL, no-observed-adverse-effect level; MRSD, maximum recommended starting dose; BSA, body surface area; CNS, central nervous system; MAD, multiple ascending dose; PK, pharmacokinetic; Vmax, maximum reaction velocity; Km, Michaelis constant; TDM, therapeutic drug monitoring; CL, clearance; fu, fraction unbound; AAG, α1-acid glycoprotein.

**Table 9:** Operational decision matrix specifying, for each compound class, the preferred method, required supporting data, key diagnostic checks, and major sources of uncertainty.

Compound Class/Scenario	Preferred Method	Required Supporting Data	Key Diagnostic Checks	Major Sources of Uncertainty
Small molecules, CYP-dominated clearance	Multi-species allometry with rule of exponents, cross-checked by IVIVE and PBPK	Multi-species in vivo CL and V; microsomal or hepatocyte intrinsic clearance; fu, p and fu, mic; blood-to-plasma ratio	Rule-of-exponents category; allometry and IVIVE agreement within about two-fold; residual inspection	Steep or shallow exponents; species CYP differences; choice of scaling factors
Transporter substrates (OATP, P-gp, BCRP, OCT/MATE)	Transporter-aware PBPK with explicit hepatic uptake and efflux	Sandwich-cultured hepatocyte uptake; transfected-cell kinetics; ECCS class; relevant pharmacogenetics	Uptake versus metabolism rate-limitation; sensitivity to transporter abundance; IVIVE under-prediction pattern	Transporter abundance and reference values; evolving consensus scaling factors
Biologics (mAbs and large proteins)	TMDD-aware PK/PD modelling; MABEL-anchored dose	Non-human primate linear-phase PK; target expression and turnover; binding constants; in vitro potency; cross-reactivity	Identifiability of linear versus TMDD parameters; MABEL versus MRSD comparison; immunogenicity risk	Poorly identifiable TMDD parameters (often fixed); anti-drug antibodies; target dynamics
Paediatric extrapolation	Paediatric PBPK with enzyme and transporter ontogeny	Verified adult PBPK model; ontogeny and maturation functions	Adult-model verification before scaling down; age-band sensitivity analysis	Ontogeny parameter gaps in the youngest groups; limited paediatric clinical data
CNS-active compounds	PBPK with brain compartment; MABEL; slow dose escalation	Unbound brain-to-plasma partition (Kp, uu); off-target lipase and kinase screens	CNS exposure versus potency margin; off-target liability flags	Brain-penetration prediction; off-target CNS effects not indexed by body weight
Non-linear or saturable PK	Michaelis-Menten parameterisation within mechanistic PBPK	Repeat-dose PK with full Vmax and Km; dose-ranging exposure	Dose-proportionality assessment; saturation at projected clinical concentrations	Extrapolation beyond the characterised concentration range
Highly protein-bound drugs (more than 99% bound)	Unbound-clearance allometry; PBPK with species-specific fu	Equilibrium dialysis at clinical concentrations; AAG levels; species fu	Reproducibility of fu measurement; divergence of unbound versus total prediction	Small fu errors amplifying unbound predictions; concentration-dependent binding

The principal methods available for interspecies pharmacokinetic prediction, together with their data requirements, strengths, limitations, and appropriate applications, are summarized in Table 10.

## 14. Limitations

Several limitations should temper the conclusions drawn here. First, predictive performance depends critically on the quality and number of preclinical species: panels of three to four species are typical but provide only a small leverage for extrapolation across the body-weight range, and the choice of species often reflects historical convention rather than mechanistic relevance to the human pathway. Second, the published allometry literature is subject to substantial publication bias toward successful predictions; compounds in which allometry failed and were re-projected by other means are systematically under-represented, thereby inflating the apparent reliability of the method [21,24]. Third, transporter-mediated clearance and uptake involve scaling assumptions about transporter abundance and activity that carry greater uncertainty than those for CYP-mediated metabolism, and consensus reference values continue to evolve [14,42]. Fourth, idiosyncratic toxicities, such as those underlying the fialuridine and troglitazone failures, are by definition not captured by any pharmacokinetic scaling framework, and even sophisticated PBPK or PBPK–PD models cannot reliably anticipate them without orthogonal mechanistic assays [69,70,75,76]. Fifth, retrospective benchmark analyses, including those underpinning the rule of exponents and many IVIVE validation studies, may not generalize to chemically novel modalities (covalent inhibitors, PROTACs, oligonucleotides, peptide-drug conjugates, RNA therapeutics) where the empirical base remains thin [6,21]. Sixth, the worked examples in Section 2.2 and Appendix A are hypothetical and constructed for didactic clarity; real datasets typically exhibit greater dispersion, more pronounced curvature, and substantial protein-binding and blood-to-plasma effects that complicate the simple log–log fit. Readers should treat the convergence between methods in our framework as a necessary but not sufficient condition for confidence in the human projection.

## 15. Future Directions

The next phase of interspecies translation is likely to be shaped less by refinement of body-weight exponents than by the convergence of mechanistic biology, computational modelling, and engineered human systems. Machine learning is already being integrated into pharmacometric workflows for parameter estimation, covariate discovery, and surrogate modelling [98]. Several developments are poised to extend this. Graph neural networks, which operate directly on molecular structure, offer a route to predicting ADME endpoints and tissue partitioning from chemistry rather than animal data, and large, pretrained foundation models spanning heterogeneous pharmacological datasets may provide transferable priors for compounds with sparse preclinical information. A recurring design tension runs through these methods: purely data-driven black-box predictors can achieve strong in-distribution accuracy but extrapolate poorly and resist mechanistic interpretation, whereas mechanistic and hybrid models trade some flexibility for transparency and regulatory defensibility [97]. For first-in-human prediction, where extrapolation beyond the observed range is the entire point, this argues for mechanism-anchored or hybrid approaches rather than unconstrained black-box fits.

Hybrid physiologically based pharmacokinetic and machine-learning systems are the most immediate prospect: ML emulators can accelerate computationally expensive PBPK simulations and impute missing system or compound parameters. At the same time, the PBPK scaffold preserves physiological interpretability, and Bayesian updating refines predictions as data accrue. Patient-specific digital twins, combining individual omics, imaging, and longitudinal exposure data with mechanistic PK/PD models, are beginning to show proof of concept in oncology and rare diseases. In parallel, microphysiological systems, organoids, and organ-on-chip platforms now generate human-cell-derived ADME and toxicity data with sufficient fidelity to displace certain animal endpoints, reducing reliance on cross-species body-weight extrapolation for the very questions allometry was invoked to answer. Across all of these, allometry is best understood not as a competitor to replace, but as a low-cost, interpretable framework to be embedded within and triangulated against richer mechanistic and computational frameworks.

## 16. Conclusions

Allometric scaling endures as a foundational tool in drug development because it imposes a biologically grounded, quantitative discipline on interspecies extrapolation. The three-quarter power relationship between metabolic rate and body mass, underpinned by the fractal geometry of vascular networks, provides a principled starting point for projecting pharmacokinetic parameters from preclinical species to humans. However, the history of translational pharmacology is replete with cautionary examples, including fialuridine, TGN1412, BIA 10-2474, benoxaprofen, terfenadine, and the toxicodynamic counterparts of thalidomide and the fenfluramine combination, demonstrating that compound-specific biology can override any scaling relationship.

The contemporary landscape offers substantially more powerful tools than simple allometry alone can provide. IVIVE provides a mechanistic bridge from in vitro enzyme and transporter kinetics to in vivo clearance. PBPK platforms embed allometric priors within organ-resolved physiological models that can simulate diverse populations, DDIs, and formulation effects. The Wajima method enables profile-level predictions complementary to parameter-based scaling. The ECCS, building on BDDCS, provides an a priori framework for predicting which scaling approach is most appropriate for a given compound’s clearance mechanism. For biologic therapeutics, target-mediated drug disposition and FcRn-mediated recycling impose additional layers of non-linearity that demand mechanism-based PK/PD models and MABEL-anchored dose selection rather than reliance on empirical allometric exponents.

This review advocates treating allometry as a disciplined starting hypothesis rather than a doctrine. Its value is maximized when integrated into a triangulation framework that compares allometric estimates with IVIVE, PBPK, MABEL, and BSA-scaled MRSD, selecting the most conservative, mechanistically supported starting dose for FIH studies. As regulatory agencies increasingly accept PBPK-based submissions and as AI- and microphysiological-systems data enter the evidence base, the future of interspecies translation lies not in any single scaling law but in the intelligent synthesis of empirical, mechanistic, computational, and human-tissue-based approaches.

## Figures and Tables

**Table 1:** Typical allometric applications and common starting exponents for pharmacokinetic parameters.

Species	Typical BW (kg)	Common PK Extrapolations	Key Considerations	References
Mouse	0.02–0.03	CL ≈ 0.75; V_D_ ≈ 1.0	High metabolic rate; rapid CL per kg	[6,15,16]
Rat	0.2–0.4	CL 0.70–0.80; V_D_ 0.9–1.0	Oral F may differ; unique CYP isoforms	[6,15,17]
Rabbit	2–4	CL 0.70–0.85; V_D_ ≥ 1.0	Coprophagy; unique GI physiology	[6,16]
Dog	8–15	CL 0.65–0.80; V_D_ 0.8–1.0	Lacks NAT2; biliary excretion differs	[6,18]
Non-human primate	2–10	CL 0.70–0.85; V_D_ 0.9–1.1	Closest to human but CYP differences exist	[6,18]
Human (FIH dose target)	60–80	Reference endpoint	Polymorphisms and DDIs can dominate	[17,19,20]

Note: Species body weights anchor expected scale; the listed exponents serve as empirical priors that should be corroborated against mechanism, enzyme/transporter expression, binding, and formulation before extrapolation [6,21]. Coprophagy is an obligate cecotrophic behavior in rabbits, with a substantial impact on enteric absorption and microbiota-mediated drug metabolism; in dogs, where coprophagia is observed, it is sporadic and behavioral rather than physiologically obligate, and is not a routine ADME determinant in drug development programs [6,18]. Abbreviations: BW, body weight; PK, pharmacokinetic; CL, clearance; V_D_, volume of distribution; F, bioavailability; CYP, cytochrome P450; GI, gastrointestinal; NAT2, N-acetyltransferase 2; FIH, first-in-human; DDIs, drug-drug interactions.

**Table 2:** Hypothetical allometric computation for clearance.

Species	Body Weight (kg)	CL (mL/min)
Mouse	0.025	0.70
Rat	0.250	6.25
Monkey	5.000	15.00
Dog	10.000	40.00
Human	70.000	(to predict)

**Table 3:** Failure-mode taxonomy of the translational case studies, classifying each by primary failure mode, predominant domain, and the evidence that could have mitigated the risk.

Case (Section)	Primary Failure Mode	Predominant Domain	Evidence That Could Have Mitigated the Risk
Benoxaprofen (Section 7.8)	Over-prediction of clearance; much slower human elimination than body-weight projection, with accumulation in the elderly	PK	Single-species and IVIVE cross-check of metabolic clearance; age-stratified PK
Cerivastatin (Section 7.7)	Metabolic and transporter drug interaction (CYP2C8 and OATP1B1 with gemfibrozil)	PK (drug interaction)	Dedicated CYP2C8 and OATP1B1 interaction studies; transporter-aware IVIVE
Terfenadine (Section 7.9)	Parent accumulation on CYP3A4 inhibition driving off-target hERG block and QT prolongation	PK leading to PD (off-target)	hERG and QT screening; CYP3A4 interaction studies; active-metabolite strategy
TGN1412 (Section 7.2)	Target- and immune-mediated cytokine release not captured by pharmacokinetics	PD (target biology); trial design	MABEL-anchored dosing; ex vivo human whole-blood cytokine assays; staggered sentinel dosing
BIA 10-2474 (Section 7.3)	Off-target toxicity emerging at supratherapeutic exposures	Toxicodynamic (off-target); trial design	Broad off-target and lipase selectivity screening; slower dose escalation
Fialuridine (Section 7.1)	Intrinsic, human-relevant mitochondrial toxicity	Toxicodynamic	Mitochondrial and longer-duration in vitro toxicity assays; mechanistically relevant species
Troglitazone (Section 7.4)	Reactive-metabolite and idiosyncratic hepatotoxicity	Toxicodynamic	Reactive-metabolite trapping and covalent-binding assays; hepatic safety biomarkers
Torcetrapib (Section 7.5)	Off-target pharmacology (aldosterone, raised blood pressure)	PD (off-target)	Secondary-pharmacology and off-target screening; mechanism-based safety pharmacology
Rofecoxib (Section 7.6)	On-target, mechanism-based class effect (COX-2 selectivity; prothrombotic risk)	PD (on-target)	Prostacyclin and thromboxane balance assessment; cardiovascular outcome surveillance

**Table 4:** Case studies of translational failure and corrective practice.

Program	Animal Signals	Human Outcome	Missing Mechanism	Corrective Practice [Ref]
Fialuridine	Reassuring preclinical profile	Catastrophic hepatic failure; 5 deaths	Human-specific mitochondrial toxicity	Stop rules; human-relevant assays [69,70]
TGN1412	Tolerability in NHPs	Cytokine storm in all volunteers	CD28 superagonism species differences	MABEL-based dosing; ex vivo human assays; sentinel dosing [54,68,71,72]
BIA 10-2474	Preclinical tolerability	Fatal neurologic injury	Off-target CNS lipase effects	Slower MAD escalation; biomarkers; imaging [73,74]
Troglitazone	Acceptable preclinical toxicology	Severe DILI; market withdrawal	Idiosyncratic mitochondrial injury	Enhanced DILI monitoring; mechanistic toxicity screens [75,76]
Torcetrapib	Biomarker improvement	Increased mortality	Off-target haemodynamics	Holistic benefit–risk beyond surrogates [25]
Rofecoxib	Animal efficacy	Increased thrombotic events	COX-2 vascular effects	Long-term cardiovascular outcome trials [77,78]
Cerivastatin	Acceptable preclinical PK	Fatal rhabdomyolysis with gemfibrozil co-use	CYP2C8 plus OATP1B1 inhibition DDI	Transporter-DDI screening; ECCS classification [41,43,79]
Benoxaprofen	Short t½ in rat and dog; standard hepatic profile	Fatal hepatotoxicity and photosensitivity, elderly enriched	Slow human glucuronidation; renal accumulation in elderly	Elderly/renal-impairment PK pre-marketing; UGT IVIVE [80,81]
Terfenadine	Extensive first-pass metabolism in animal species; low parent exposure	Torsades de pointes with CYP3A4 inhibitor co-use	Parent hERG block unmasked by CYP3A4 DDI	hERG and thorough QT studies; metabolite-linked PBPK DDI [82,83]

Note: Each row links a preclinical program to the human outcome and the missing mechanism, motivating conservative MRSD/MABEL approaches, human-relevant assays, and escalation designs that can safely interrogate human pharmacology. Abbreviations: NHPs, non-human primates; MABEL, minimum anticipated biological effect level; MAD, multiple ascending dose; CNS, central nervous system; DILI, drug-induced liver injury; COX-2, cyclooxygenase-2; PK, pharmacokinetics; CYP2C8, cytochrome P450 2C8; OATP1B1, organic anion transporting polypeptide 1B1; DDI, drug-drug interaction; ECCS, Extended Clearance Classification System; t½, elimination half-life; UGT, uridine 5′-diphospho-glucuronosyltransferase; IVIVE, in vitro-in vivo extrapolation; hERG, human ether-à-go-go-related gene; QT, QT interval; PBPK, physiologically based pharmacokinetic.

**Table 5:** Body-surface-area K_m_ factors and illustrative HED to MRSD conversion.

Species	K_m_	NOAEL (mg/kg)	HED (mg/kg) = NOAEL × (K_m_, sp/K_m_, hu)	Safety Factor	MRSD (mg) at 70 kg
Mouse	3	100	8.11	10	56.8
Rat	6	50	8.11	10	56.8
Dog	20	20	10.81	10	75.7
Monkey	12	15	4.86	10	34.0
Human (ref)	37	—	—	—	—

Note: The most conservative species (lowest MRSD) should generally govern FIH dose selection. The MRSD should be triangulated with MABEL and PBPK-based exposure predictions [12,16,59]. Abbreviations: K_m_, body weight conversion factor; NOAEL, no-observed-adverse-effect level; HED, human equivalent dose; sp, species; hu, human; MRSD, maximum recommended starting dose; ref, reference.

**Table 6:** Planning matrix for pediatrics, organ impairment, and drug–drug interactions.

Scenario	Key Assumptions	Expected Impact on AUC/C_max_	Dose/PK Strategy [Ref]
Paediatrics (1–12 y)	Enzyme/transporter maturation; altered body composition	AUC may increase or decrease by pathway	Weight-based dosing with maturation functions; paediatric PBPK [22,87,88,89]
Renal impairment	Reduced GFR; transporter alterations	AUC increase for renally cleared drugs	eGFR-based dose bands [90]
Hepatic impairment	Decreased CL_int_ and hepatic blood flow	AUC increase; t½ prolongation	Child–Pugh-guided dosing; hepatic PBPK [22]
Strong inhibitor DDI	Enzyme/transporter blockade	AUC increase; safety risk	Contraindication or dose reduction; TDM [22,85]

Note: Population PBPK models embed age-, genotype-, disease-, and perpetrator-specific physiology and, when externally verified, can justify label recommendations for drug–drug interactions (DDIs), organ impairment, and pediatric bridging. Abbreviations: AUC, area under the concentration-time curve; C_max_, maximum plasma concentration; PK, pharmacokinetic; PBPK, physiologically based pharmacokinetic; GFR, glomerular filtration rate; eGFR, estimated glomerular filtration rate; CL_int_, intrinsic clearance; t½, elimination half-life; DDI, drug-drug interaction; TDM, therapeutic drug monitoring.

**Table 7:** Neutral comparison of the PBPK and PK platforms discussed, mapping principal scope and context of use to the corresponding evidentiary expectation [95,96].

Platform (Developer)	Principal Scope and Typical Context of Use	Representative Evidentiary Expectation
GastroPlus (Simulations Plus)	Mechanistic oral absorption and formulation (ACAT); whole-body PBPK; DDI and special-population simulation	Verification against observed clinical PK; fit-for-purpose qualification proportionate to the context of use
Simcyp (Certara)	Population PBPK with virtual populations; DDI, paediatric and organ-impairment simulation	Documented system-parameter provenance; sensitivity analysis; verification appropriate to the regulatory question
PK-Sim/MoBi (Open Systems Pharmacology; open source)	Whole-body PBPK (PK-Sim) and customisable multiscale modelling (MoBi)	Same verification standards; open model code supports transparency and reproducibility
Phoenix WinNonlin (Certara)	Non-compartmental and population PK; empirical allometric fitting (not a mechanistic PBPK platform)	Standard NCA and population-PK reporting; allometric outputs treated as priors rather than stand-alone evidence

**Table 10:** Comparison of the principal interspecies prediction methods, the data they require, and their typical use.

Method	Data Required	Strengths	Weaknesses	Best Use
Simple allometry	Multi-species in vivo PK (CL, V)	Fast, transparent, minimal data	Ignores species-specific ADME; exponent not universal	Early screening and first-pass CL and V estimates
Rule of exponents (ROE)	Multi-species in vivo PK plus maximum life-span or brain-weight correction	Improves clearance prediction by correcting steep exponents	Empirical correction; unreliable for high-extraction or transporter-driven drugs	Small-molecule clearance when simple allometry over- or under-predicts
IVIVE	In vitro metabolism (microsomes, hepatocytes), binding and physicochemical data	Mechanistic; resolves species differences in metabolic capacity	Sensitive to in vitro artefacts and choice of scaling factors	Hepatically cleared small molecules; metabolic clearance prediction
PBPK	Physicochemical, in vitro ADME and system physiology parameters	Comprehensive; supports DDI, organ impairment and paediatric extrapolation	Data- and labour-intensive; many parameter assumptions	Regulatory submissions and special-population prediction
Wajima profile superposition	Multi-species concentration–time profiles	Recovers the full human concentration–time profile, not only point parameters	Assumes interspecies profile similarity after normalization	Exposure and profile prediction when curve shape, not only clearance, matters

## Data Availability

No new datasets were generated for this review. All numerical examples are illustrative and constructed from values disclosed in the cited literature; the worked clearance example in Section 2.2 and the worked Wajima example in Section 6.2 use hypothetical input values for didactic purposes and do not reflect proprietary data. The underlying regression statistics, residuals, and multi-species pharmacokinetic parameters used to generate the figures are provided in full in Appendix A.

## Abbreviations

The following abbreviations are used in this manuscript:

AAG	Alpha-1-acid glycoprotein
ACAT	Advanced Compartmental Absorption and Transit
ADAM	Advanced Dissolution, Absorption and Metabolism
ADME	Absorption, Distribution, Metabolism, and Excretion
AIC	Akaike Information Criterion
AUC	Area Under the Curve
BCRP	Breast Cancer Resistance Protein
BDDCS	Biopharmaceutics Drug Disposition Classification System
BIC	Bayesian Information Criterion
BMR	Basal Metabolic Rate
BSA	Body Surface Area
BrW	Brain Weight
CL	Clearance
CL_int_	Intrinsic Clearance
CNS	Central Nervous System
CRBN	Cereblon
CYP	Cytochrome P450
C_max_	Maximum Plasma Concentration
DDI	Drug–Drug Interaction
DILI	Drug-Induced Liver Injury
DNA	Deoxyribonucleic Acid
EC10	10% Effective Concentration
ECCS	Extended Clearance Classification System
EMA	European Medicines Agency
FAAH	Fatty Acid Amide Hydrolase
FIAU	Fialuridine
FIH	First-in-Human
FcRn	Neonatal Fc Receptor
GFR	Glomerular Filtration Rate
HED	Human Equivalent Dose
hERG	Human Ether-à-go-go-Related Gene
ICH	International Council for Harmonisation
IV	Intravenous
IVIVE	In Vitro–In Vivo Extrapolation
Kp, uu	Unbound Brain-to-Plasma Partition Coefficient
Km	Body Weight to Body Surface Area Conversion Factor
LBF	Liver Blood Flow
MABEL	Minimum Anticipated Biological Effect Level
MAD	Multiple Ascending Dose
MATE	Multidrug and Toxin Extrusion Transporter
ML	Machine Learning
MLP	Maximum Life-Span Potential
MRSD	Maximum Recommended Starting Dose
MRT	Mean Residence Time
NAT2	N-acetyltransferase 2
NHP	Non-Human Primate
NOAEL	No-Observed-Adverse-Effect Level
OATP	Organic Anion-Transporting Polypeptide
OCT	Organic Cation Transporter
OLS	Ordinary Least Squares
P-gp	P-glycoprotein
PBPK	Physiologically Based Pharmacokinetic
PD	Pharmacodynamic
PK	Pharmacokinetic
PK/PD	Pharmacokinetic/Pharmacodynamic
PROTAC	Proteolysis-Targeting Chimera
QSP	Quantitative Systems Pharmacology
QT	QT interval
ROE	Rule of Exponents
t½	Elimination Half-life
TMDD	Target-Mediated Drug Disposition
UGT	Uridine 5′-diphospho-glucuronosyltransferase
V_D_	Volume of Distribution
Vmax	Maximum Reaction Velocity
V_ss_	Volume of Distribution at Steady State
fu	Fraction Unbound
mAbs	Monoclonal Antibodies

## Appendix A. Supporting Numerical Work

This appendix provides the underlying numerical work supporting the analyses in Section 2 and Section 6. It contains the source data, ordinary least squares regression statistics, and per-species residuals for the worked example of Section 2.2 (Figure 1), together with the multi-species pharmacokinetic parameters and exposure dispersion underlying the Wajima worked example of Section 6.2 (Figure 3).

### Appendix A.1. OLS Fit for the Worked Example of Section 2.2

Ordinary least squares (OLS) regression of log_10_(CL) on log_10_(BW) for the four species in Table 2 of the manuscript confirmed the fit is log_10_(CL) = 0.603 × log_10_(BW) + 0.931, R^2^ = 0.939.

#### Appendix A.1.1. Source Data

**Table A1:** Source preclinical clearance data (the same four species used in Table 2 of the manuscript) are shown with their log_10_-transformed values.

Species	Body Weight (kg)	Clearance (mL/min)	log_10_ BW	log_10_ CL
Mouse	0.025	0.70	−1.6021	−0.1549
Rat	0.250	6.25	−0.6021	+0.7959
Monkey	5.000	15.00	+0.6990	+1.1761
Dog	10.00	40.00	+1.0000	+1.6021

#### Appendix A.1.2. OLS Regression Statistics

**Table A2:** OLS regression statistics for the worked example in Section 2.2.

Statistic	Value	Standard Error
Slope (allometric exponent, b)	0.6029	0.1083
Intercept (log_10_ a)	0.9309	0.1138
Coefficient of determination, R^2^	0.9394	—
Residual sum of squares (log units^2^)	0.07882	—
Number of preclinical species, *n*	4	—
Predicted human CL at 70 kg	110 mL/min	—

Note: a, proportionality constant (intercept of the allometric equation); b, allometric exponent (slope of the log-log regression); R^2^, coefficient of determination; n, number of preclinical species; CL, clearance; OLS, ordinary least squares.

#### Appendix A.1.3. Per-Species Residuals

**Table A3:** Residuals from OLS fit.

Species	log_10_ BW	log_10_ CL Observed	log_10_ CL Fitted	Residual
Mouse	−1.6021	−0.1549	−0.0350	−0.1200
Rat	−0.6021	+0.7959	+0.5679	+0.2279
Monkey	+0.6990	+1.1761	+1.3523	−0.1762
Dog	+1.0000	+1.6021	+1.5338	+0.0682

Note: The rat residual is the largest in absolute terms (+0.228 log units, equivalent to a 1.69-fold deviation), consistent with the well-described high hepatic blood flow per kg and elevated CYP2C11/CYP2C12 activity in the rat.

#### Appendix A.1.4. Equation and Human Projection

The regression equation is:

*log_10_*(*CL*) *=* 0.603 × *log*_10_(*BW*) + 0.931 (A1)

Equivalently, in the multiplicative form:

*CL* = 10^0.931^ × *BW*^0.603^ = 8.53 × *BW*^0.603^ (mL/min, *BW* in kg) (A2)

For an adult human of body weight 70 kg, log_10_(70) = 1.845:

*log*_10_(*CL_human*) = 0.603 × 1.845 + 0.931 = 2.043 (A3)

*CL_human* = 10^2.043^ = 110 mL/min (A4)

#### Appendix A.1.5. Prediction Uncertainty at the Human Body Weight

The point estimate of 110 mL/min in Section 2.2 conveys none of the uncertainty attached to a four-species extrapolation carried well beyond the observed body-weight range. Using the ordinary least squares fit of Appendix A.1.2 (slope 0.6029, intercept 0.9309, residual standard error 0.1985 log_10_ units on two degrees of freedom), the standard error of the fitted log_10_ clearance at a human body weight of 70 kg (log_10_ 70 = 1.8451) is 0.2122 log units, and the standard error for a single new prediction is 0.2906 log units.

Applying the two-tailed t value for two degrees of freedom (t = 4.303), the 95% confidence interval for the mean predicted log_10_ clearance is 1.130 to 2.956, equivalent to approximately 13 to 905 mL/min. The 95% prediction interval for a new observation is 0.793 to 3.294, equivalent to approximately 6 to 1970 mL/min. These intervals span about two orders of magnitude despite an R^2^ of 0.939, quantitatively reinforcing the argument of Section 2.3 that goodness of fit within the preclinical range is not a measure of extrapolative reliability. The bands are reported numerically rather than shown in Figure 1, where their widths would compress the fitted line and the four data points to the point of visual indistinguishability.

### Appendix A.2. Wajima Multi-Species Worked Example

An example with mouse, rat, monkey, and dog (the same species set as Section 2.2 of the manuscript) after a 1 mg/kg IV bolus, using plausible allometric values for CL/kg and V_ss_/kg, is presented. The clearance values used in this Wajima example are an independent hypothetical parameter set chosen to illustrate the superposition principle and are not numerically linked to those in Section 2.2 or Appendix A.1. The example demonstrates that profiles which look markedly different on absolute axes collapse onto a single common curve after Wajima normalization (rescaling time by MRT and concentration by Dose/V_ss_), which is the visual signature of the method.

#### Appendix A.2.1. Per-Species PK Parameters

**Table A4:** Multi-species pharmacokinetic parameters used in the Wajima worked example.

Species	BW (kg)	CL/kg (mL/min/kg)	V_ss_/kg (L/kg)	CL (mL/min)	V_ss_ (L)	MRT (min)	Dose/V_ss_ (mg/L)
Mouse	0.025	90.0	1.50	2.25	0.0375	16.7	0.667
Rat	0.250	50.0	1.00	12.5	0.250	20.0	1.000
Monkey	5.00	25.0	0.80	125	4.00	32.0	1.250
Dog	10.0	18.0	0.70	180	7.00	38.9	1.429

Note: CL/kg and V_ss_/kg decline modestly with body size in a pattern consistent with empirical observation across small molecules with hepatic CYP-mediated clearance. MRT (=V_ss_/CL) spans 16.7–38.9 min (a 2.3-fold range) and Dose/V_ss_ spans 0.67–1.43 mg/L (a 2.1-fold range), driving the visible separation of the absolute profiles.

#### Appendix A.2.2. Exposure-Metric Dispersion in Absolute vs. Normalized Space

**Table A5:** Dispersion of total exposure (AUC) and peak concentration (C_max_) across species.

Species	AUC_0_–∞ Absolute (mg·min/L)	C_max_ Absolute (mg/L)	AUC_0_–∞ Normalized	C_max_ Normalized
Mouse	11.1	1.13	1.00	1.70
Rat	19.9	1.70	1.00	1.70
Monkey	39.8	2.13	1.00	1.70
Dog	55.3	2.43	1.00	1.70
Fold range (max/min)	5.0×	2.1×	1.0×	1.0×

Note: In absolute units, AUC spans a 5.0-fold range and C_max_ a 2.1-fold range. After Wajima normalization, the dispersion collapses to a 1.0-fold range, with the four species becoming indistinguishable. This is the operational definition of superposition that motivates the method.

#### Appendix A.2.3. Notes on Assumptions

1. CL/kg and V_ss_/kg were assumed to fall modestly with body size, broadly consistent with values for representative small molecules with hepatic CYP-mediated clearance. Specific compound values would replace these in a real application.
2. The shape function f(τ) was chosen as a biexponential with a distribution phase (α = 4.0) and an elimination phase (β = 0.625), normalized so that the integral over (0, ∞) equals one. This corresponds to a typical 2-compartment IV-bolus profile.

The dose was set at 1 mg/kg in each species to match the body-weight-normalized convention used in Section 2.2.
